# Julius – a template based supplementary electronic health record system

**DOI:** 10.1186/1472-6947-7-10

**Published:** 2007-05-02

**Authors:** Rong Chen, Gösta Enberg, Gunnar O Klein

**Affiliations:** 1 Department of Microbiology, Tumor and Cell Biology, Karolinska Institutet, Stockholm, Sweden; 2 Department of Neurobiology, Care Sciences and Society, Karolinska Institutet, Stockholm, Sweden; 3 Department of Medicine, Karolinska Institutet, Stockholm, Sweden

## Abstract

**Background:**

EHR systems are widely used in hospitals and primary care centres but it is usually difficult to share information and to collect patient data for clinical research. This is partly due to the different proprietary information models and inconsistent data quality. Our objective was to provide a more flexible solution enabling the clinicians to define which data to be recorded and shared for both routine documentation and clinical studies. The data should be possible to reuse through a common set of variable definitions providing a consistent nomenclature and validation of data. Another objective was that the templates used for the data entry and presentation should be possible to use in combination with the existing EHR systems.

**Methods:**

We have designed and developed a template based system (called Julius) that was integrated with existing EHR systems. The system is driven by the medical domain knowledge defined by clinicians in the form of templates and variable definitions stored in a common data repository. The system architecture consists of three layers. The presentation layer is purely web-based, which facilitates integration with existing EHR products. The domain layer consists of the template design system, a variable/clinical concept definition system, the transformation and validation logic all implemented in Java. The data source layer utilizes an object relational mapping tool and a relational database.

**Results:**

The Julius system has been implemented, tested and deployed to three health care units in Stockholm, Sweden. The initial responses from the pilot users were positive. The template system facilitates patient data collection in many ways. The experience of using the template system suggests that enabling the clinicians to be in control of the system, is a good way to add supplementary functionality to the present EHR systems.

**Conclusion:**

The approach of the template system in combination with various local EHR systems can facilitate the sharing and reuse of validated clinical information from different health care units. However, future system developments for these purposes should consider using the openEHR/CEN models with shareable archetypes.

## Background

Electronic health record (EHR) systems are widely used in hospitals and primary care centres in the Stockholm County Council. Essentially all primary care records and recently almost all hospital medical records are stored electronically in this region. However, within the region there are over twenty-five different EHR systems in use.

Since different vendors are using both different proprietary information models and different terminologies when recording the (clinical) data, it's nearly impossible to collect consistent and validated data into centralized databases for further analysis. There are also more than 50 national quality registries in Sweden, collecting patient data from health care units for quality control and clinical research, which have similar problems with different information models and terminologies.

One of these quality registries is the National Diabetes Registry (NDR) which recommends the health care units responsible for the care of diabetes patients, to report data back to the central NDR server periodically or at least once a year. The NDR service provides two ways to do this reporting, a) through a web based graphic user interface or b) through a system integration interface implemented as web services. The required format includes a list of predefined variables which needs to be mapped to the local variables within EHR system, before transferring the data automatically.

Since the integration to the NDR service is not fully supported by all of the EHR system, many clinicians have to re-enter the data manually. This procedure increases both the workload of the clinicians and the risks of introducing input errors. As a consequence the data will sometimes not be reported due to the lack of a more efficient routine. Another aspect is that the different EHR systems also utilize different validation rules for data entry, which means that the quality of recorded data might be insufficient.

Nearly all existing EHR systems are built with an explicit domain model– a common approach in current EHR software development practice. This means that the hard coded medical domain knowledge in the system results in higher cost when new requirements in clinical documentation routines occur. For example, clinicians who want to record extra (new) data to improve clinical research will have to ask the local EHR vendor to implement this new feature. Apart from the waiting time for the implementation etc, they also may have to pay extra money for the new feature. If a research program requires a large amount of data to be collected from different clinical sites (using different EHR systems), this make it much more difficult to add a specific features so the collected data will be comparable. At present it is therefore difficult to collect data regarding clinical observations and treatments in order to facilitate clinical research and quality control utilizing the current EHR systems.

The lack of integration between the different EHR systems is not only an obstacle for a more effective clinical research, but it is also a fact that may lead to a suboptimal care for the individual patient, including potential safety problems and an unnecessary waste of resources.

## Methods

### Design considerations

The new system should be able to record data both from clinical routine documentation and experimental studies. The system should be possible to integrate with the local EHR systems in clinical environment, preferably using the same Graphical User Interface (GUI) as the existing EHR system. The users should not be forced to duplicate the data that already might exist in the local EHR system. The system should be flexible enough to allow certain authorized users to define what kind of (new) data items to be recorded during either clinical routine documentation or clinical trials based on expertise in the medical domain. The users should also be able to specify the data type of the variable to be captured and its valid range to minimize the possibility of an erroneous entry.

Optionally the user should be able to link the defined data item to an external terminology, which later can be utilized for possible data aggregation and classification purposes. All the definitions should be possible to maintain directly by the medical domain experts without any involvement of the software developer(s).

More importantly, such definitions should be shared and reused by other users than the original author for data recording so that the semantics and quality of the data collected from different health care units with various EHR systems can be maintained.

### System description

We have designed and developed a template based supplementary EHR system called Julius, which allows the clinicians to define data items they want to record and then design the layout of a template that guides the clinical user when recording the data. Templates and variables can be defined during system runtime to adapt the system to meet new requirements on data recording.

**Figure 1:**
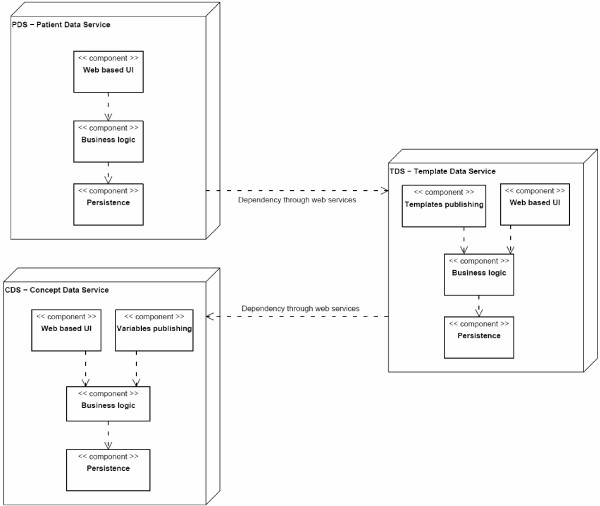
System Architecture Overview.

The system conceptually consists of three subsystems: the Concept Data Service (CDS) where variables are defined, the Template Data Service (TDS) for defining data entry templates, and Patient Data Service (PDS) where the patient data is stored and managed (Figure 1).

These three subsystems are communicating using web services. The system can be deployed according to different operational strategies depending on the requirements of the organisation. For instance the definition of variables and templates can be shared among different health care units having separate Patient Data Services. However, at least one instance of CDS must be online for TDS to work properly; similarly at least one instance of TDS must be online for PDS to run.

For the implementation in the Stockholm County Council, it was decided to keep only one centrally managed CDS, one TDS for each hospital and one PDS for each health care unit.

### Concept Data Service (CDS)

The CDS is the starting point for the medical domain experts wanting to design a template. They can define the data items corresponding to individual clinical concepts within a clinical process, for which they want to record data. For each concept, a variable definition is made and can later be shared and re-used by several other templates if needed.

The variable definition within the system consists of a) a concise term describing the concept, b) a data type of the value to be captured, c) a label for display and d) a description in plain text format. The possible data types are integer, decimal, date, text and pre-defined value sets. An optional unit designator can be assigned to the variable if it is necessary to represent a quantity value.

**Figure 2:**
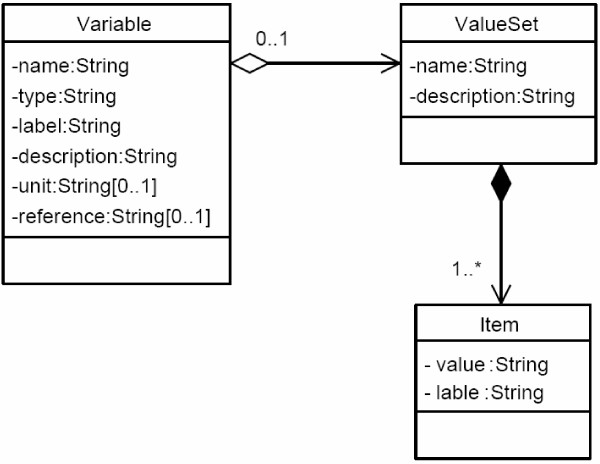
CDS UML Class Diagram.

External codes from standard terminologies can be linked to a variable definition as a reference term usually done by a specialist of the reference terminology. Depending on the nature of the terminology, the reference term can be used to perform things like classification (e.g. with ICD10 code) and aggregation (e.g. with SNOMED CT code) of clinical data. Figure 2 shows the information model of the core part of the CDS variable definition. All variable definitions will by default be inactive when first created by the variable author, and not ready for use until the (new) definition has been approved by a CDS administrator (medical domain expert). This procedure was chosen in order to ensure that all definitions maintain a good quality and consistency. The administrator is also responsible to ensure that new versions of variable definitions do not cause problems in templates where they are already in use. After the approval of the CDS administrator the definition is activated and can be use by the various templates. A variable definition can also at any time be deactivated for different reasons, such as maintenance. A deactivated variable definition will in any case not be available for use when composing a new template but can be used for displaying previously entered data.

In order to find the appropriate variable definitions when composing new templates, a dynamic category structure was implemented in the CDS. All variables and value sets are associated with a specific category, representing an organization or clinical speciality. The users can easily navigate to their own sub-category to manage their variables. A global search function is also available for the variable definitions based on their attributes.

**Figure 3:**
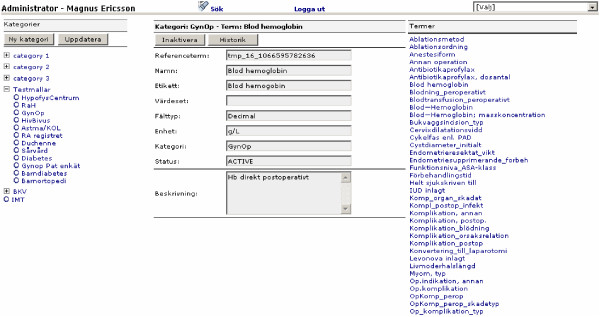
CDS Administration screen.

The user interface of the CDS is web based and accessible through an ordinary web browser (Figure 3). Multiple users can work at same time and all definitions are stored centrally on the CDS server. Upon activation, the new variable definitions become immediately available through the web services interface. The definitions are expressed in XML format, which itself is constrained by a predefined XML Schema.

### Template Data Service (TDS)

**Figure 4:**
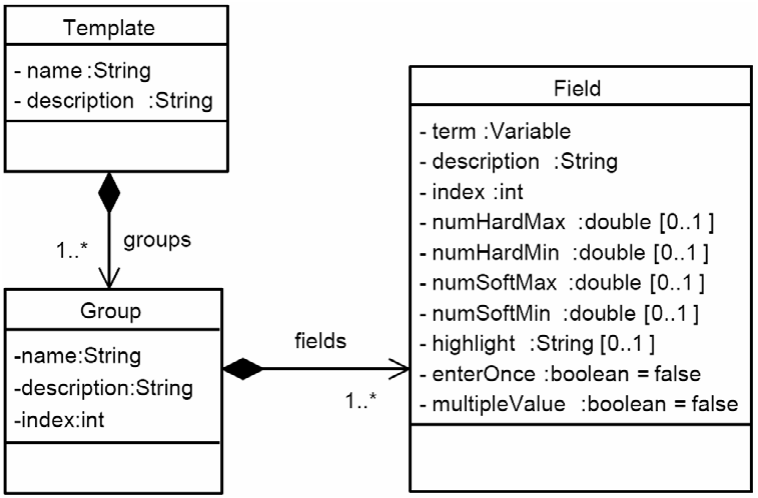
TDS UML Class Diagram.

The TDS subsystem allows users to compose new templates or edit old ones that utilize the variable definitions published by the CDS. The template definitions are later used by the PDS subsystem to read and record the patient data. A template consists of a list of groups, which in turn consists of fields that refer to the variable definitions with optional validation rules. Figure 4 shows the information model of the core part of the TDS.

Users can within a template create groups to logically organize the variables according to the specific characteristics of the clinical concepts. The groups determine the layout of the user interface for data entry and display. This simple yet intuitive approach enables the clinical users themselves to adjust the system to meet their specific needs.

For example, a user can choose to enter data from laboratory results, clinical findings and treatment decisions into three different groups with the names "Lab Result", "Diagnosis" and "Treatment" respectively. The groups can also be modified to add new variable definitions or remove existing variable definitions if required.

In the case of integer and decimal data type variables, minimal and maximal values can be specified for validation purposes. It has to be noticed that the different validation rules and descriptions can be specified for the same variable used in different templates. One advantage of this specialisation in the templates is that the same variable may have different limits in different contexts, e.g. the limits of body weight certainly differ in the context of adults and children. Furthermore, the template author can provided textual description to overwrite the original description supplied by the variable definition author. It is expected that such descriptions would give helpful information when data are being recorded, for instance some explanation of the usage of a customized term. The definition of a variable is thus more reusable than that of a template.

**Figure 5:**
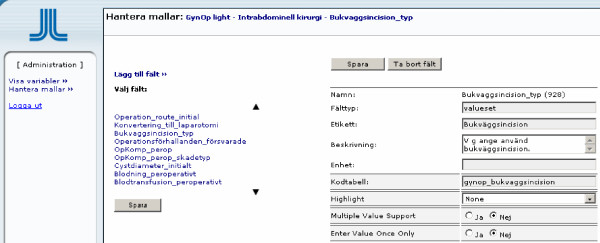
TDS administration screen.

As in the case for the variable definitions, templates are authored, modified and activated through a web based user interface (Figure 5) and all templates are published through a web services interface (Figure 1). An XML Schema is again used to specify the XML format used to describe the templates.

### Patient Data Service (PDS)

**Figure 6:**
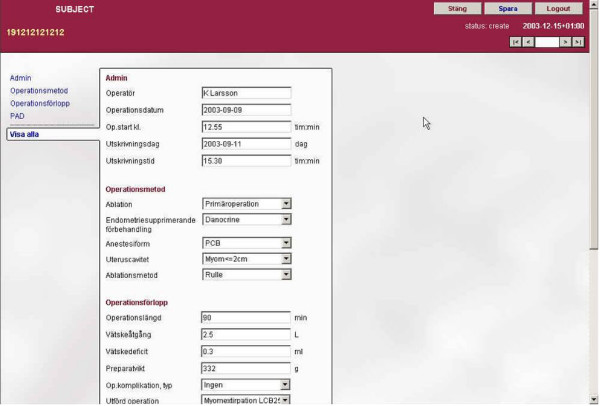
PDS Data Entry Screen.

The PDS is managing the display, editing and storage of the collected patient data. The PDS utilizes the template definitions published by TDS and transform the template into web forms ready for data entry. It also applies validations rules defined by both CDS and TDS when it receives data submitted by the user. An easy navigation is provided by the use of different groups of variables and a summarized view of the data. The web based interface (Figure 6) can be integrated with the major EHR systems deployed at several hospitals and primary care centres.

The user doesn't need to leave the local EHR client interface to be able to enter the interface of the system. A browser window with the web forms are loaded directly from the EHR system. The authentication and selection of the patient identity and relevant templates are automatically taken care by the integration mechanism between the Julius system and the local EHR client.

The data recorded through the web form are stored on the (central) PDS server, which is prerequisite for reporting to a number of national quality registries or exporting to other secondary databases dedicated to clinical research. Most of the data need to be collected are from multiple departments and health care units, which already has different EHR clients from various vendors for daily routine use. But none of the existing EHR products can offer such dynamic configuration as this dedicated template system to meet the changing requirement from clinical process and research activities. More importantly, this template system provides a way for participating members – hospitals and health care units, to define, agree upon and share terms and templates used for data recording, which leads to more consistent, more reliable clinical data to be collected. That was exactly the primary goal of this research.

### Design and implementation

The system is designed according to modern system architecture with three principal layers [1]. The presentation layer is purely web-based, which allows seamless integration with existing EHR products. The domain layer consists of a template and term engine, transformation logic and validation logic. The data source layer utilizes an object relational mapping tool and a relational database.

The system is implemented in the JAVA programming language with certain APIs from its enterprise extension J2EE. The presentation layer is implemented in HTML and JavaScript, which allows high portability among mainstream web browsers. The user interface is the result of an XSLT transformation on XML data generated by the domain layer. The domain logic is organized as a domain model, a small number of simple but coherent classes.

Several well-known open source tools and libraries are used instead of other proprietary software products, for several reasons such as lower cost, overall better openness and reliability. The open source software used in the Julius system are JBoss (application server), Hibernate (OR mapping tool), MySQL (database management system), Axis (web services library) and Xalan (XSLT library). The production system is running on the Red Hat Linux Enterprise edition.

The Julius system source code is available for review [see additional file 1].

## Results

The first release of Julius system has been deployed and integrated with Swedestar, an EHR system for the primary healthcare, in a pilot study with two primary care centres. For the follow-up of patients with diabetes a template with 50 variables has been created for the collection of clinical data. During two years, over 250 patients have been recorded with the Julius system and reported to the National diabetes registry.

The second release of the Julius system, with an enhanced template engine and an improved user interface has been deployed at Department of Infectious Diseases, at the Karolinska University Hospital. This release will later be used for Swedish HIV registry, which when fully implemented will receive input from 37 clinical units all over Sweden.

Pilot user groups have been interviewed regarding their early experience of using the Julius system. The feedback from clinicians has been positive since the Julius system empowers them in deciding what and how the clinical information should be collected. The users can define validation rules and options for the recorded concepts, which results in higher quality and more consistent patient data to be gathered for quality control and clinical research. Since the template system can be invoked seamlessly from several major EHR products, it does not change the way clinicians are used to work, nor does it force them to repeat the recording of data for clinical and/or research purposes. The web based solution of Julius allows data from different units to be collected from the same user interface regardless of the EHR software that the clinicians actually use.

## Discussion

The use of templates in combination with user defined variables with the functionality of the template system was new to the current EHR products used in the Stockholm region. The template system provides the clinical user groups with a set of tools to author variable definitions and templates, and to share the definitions of both, which alleviates the interoperability problem simply because the definitions are explicit and readily accessible. This is contrary to most of proprietary systems whose domain entities are hidden and often embedded in programming logic.

A second important aspect of the template system is that it is a highly flexible and adaptive system since its runtime behaviour is driven by templates and variable definitions created and maintained by the end users, the medical professionals themselves. Requirements unforeseen by the software developers can be easily accomplished by creating new or updating existing templates and variables. Compared with the traditional approach, the clinical users would have to require a change from the vendors and wait for the vendors to do the implementation. Not only does this take a long time and resources, but also increase the possibility of system errors. Furthermore, the traditional software engineering process requires knowledge transfer from the medical domain experts to the software engineers, which sometimes inadvertently leads to misunderstandings and new errors in the software. Given the changing nature and the complexity of medical knowledge, the driving force to adapt EHR systems has to be the medical professionals who have best understanding of the clinical procedures and what needs to be recorded. Thus the system offers a useful complementary solution to the existing EHR systems for both clinical routine and research use.

Since the PDS data are stored separately from the local EHR, there is a risk that duplicated data could be entered into both systems thus causing inconsistency of the data. It has been recommended to the users that templates that overlap with the local EHR systems should not be created and used for data entry to avoid data coherence problems.

### Other similar approaches

The OpenSDE [2] system developed by Renske K. Los et al used row-modelling technique to allow generic structured data entry. The flexibility of the OpenSDE system is similar to Julius though they are based on different solutions. Compared with the Julius approach, the OpenSDE system doesn't seem to be able to share the definitions of templates and concepts, nor does it can be integrated into existing local EHR system for the purpose of shared EHR data among different sites.

Yamazaki etc [3] reported using XML for sharing Electronic Patient Record (EPR) screen templates in EPR platform independent way. Although it's partly like what the Julius Template Service does, their focus wasn't to share EHR data but the screen templates used for structured data entry.

Other authors [4,5] have also reported template based EHR system and platform independent structured data entry, but none of these researches have the reached goal for sharing EHR data between different EHR sites.

### High level comparison to openEHR approach

It should be noted that the Julius approach is quite similar to the archetype concept described by Beale [6] in the way that Julius template is roughly a clinical domain archetype as described by Beale. Essentially, both approaches use a small set of predefined information models, which allows unlimited ways of combination to meet the changing requirements, but constrained by user defined knowledge components during system runtime. Both share the attribute of being highly flexible and adaptive; however there are some differences between these two approaches.

The archetype concept has received a wide recognition and been adopted as a key component by the openEHR foundation [7], a non-profit organization which promotes comprehensive and interoperable EHR by producing open standards and open source software. A large portion of the openEHR specifications have been incorporated into the latest draft European standard for EHR communication prEN 13606. In fact, the Archetype Definition Language (ADL), the expression form of archetypes, has been included in the part 2 of EN 13606. Also two EHR related ISO documents, namely the Technical Report of EHR Definition, Scope and Context and Technical Specification of Requirement of EHR Architecture, have been much influenced by the openEHR work. Conformance to international standards is perhaps most distinctive difference between Julius system and openEHR. The Julius system is based on a proprietary design, while the openEHR designs are publicly available specifications and based on the latest formal European standard for EHR communication.

The second key difference lies in the expressiveness of the information models. The openEHR reference information model seems to be more comprehensive and well-thought (the detailed comparison is included below). The openEHR design has been refined through more than 10 year experience from several European EHR projects and early implementations conducted both in Australia and UK. The openEHR reference model covers not only the EHR information model but also related areas like demographic data, version control, security and access control and medico-legal concerns which are not covered by the Julius model.

On the other hand, the Julius system was designed to be a complementary solution to existing EHR systems with specific goals under practical constraints from both time and resources. So it is understandable that the Julius design is not as sophisticated as that of the openEHR, which aims for full-blown and possibly future-proof EHR systems. Although openEHR was founded in 2000, the Release 1.0 of the design specifications has just been finalized at the time of writing due to continuous effort on harmonizing between openEHR and major international information model standards, e.g. HL7 RIM v3 and the CEN [8] work on EN 13606 EHR Communication.

The openEHR specifications have been validated by at least one reference implementation which is also released as open source software. Since the openEHR design is fundamentally based on the same approach as Julius – two-level modelling and its specifications are freely available and relatively well-known in the EHR world, it is worthwhile to make a more detailed comparison between the design of openEHR and Julius.

### More detailed comparison to openEHR archetypes

The Julius Variables and Templates serve similar purposes as the openEHR Archetypes. More specifically, the Julius Variables are somewhat like the leaf level constraints of the Archetypes, which essentially select input data types, predefined values and provide binding to external terminologies. The Julius Templates are equivalent to Archetypes constraining on the level of data structures and EHR compositions and entries by including a collection of Variables and grouping them according to the semantics of the screen forms. Archetypes are domain content models that can be used not only to derive screen forms but also to facilitate generic query interfaces and to build messages for exchange between different EHR systems. Julius Variables and Templates are less sophisticated by design and have limited scope compared with archetypes.

The Julius Variable only allows binding between a variable and a single terminology or coding system, and does not provide possibility to reference external terminologies for value set items. In contrast the openEHR Archetype allows binding between an archetype node to any number of target terminologies both on the entry node level and value selection level.

Interestingly the Julius Templates can also provide extra validation rules, description text besides the ones from the Variable definition, which can be regarded as local customization of the Variable constraints. They also give hints for rendering the graphic interface, e.g. with highlight flags.

**Figure 7:**
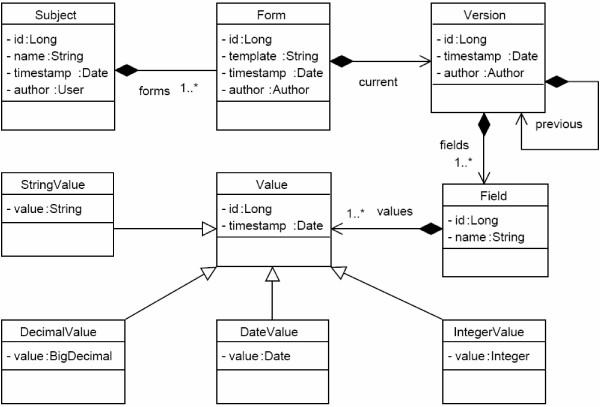
PDS UML Class Diagram.

The constraints from Julius Variables and Templates are captured in their own models and used when PDS objects are constructed. The mapping between the constraints and the PDS model are not explicit, for instance a Variable constraint is used to create an instance of Value object from PDS model (Figure 7), and a Template constraint is used to create PDS Form instance. This type of loose coupling between the constraints and the target information model is a design feature of the Julius system. The constraint model is generic in the sense that any existing EHR systems might use the Variable and Template definitions to achieve some level of interoperability without any coupling to the Julius PDS. On the other hand, without referencing the target information model, there is no guarantee that the target object instance would obey any of the constraints because it is up to the target system to map them to its internal information model. Since the constraints from Julius Variables and Templates are about common data types, limited data structures and rather simple validation rules, it should in principle be quite straightforward to map the constraints to the internal data model of any existing EHR system.

The openEHR Archetype Model is also generic, but the constraints are expressed explicitly on a selected information model, e.g. openEHR Information Model or EN 13606 Information Model. This explicit use of a target information model is lacking in Julius Variables/Templates as mentioned above. The explicitness of the Archetypes based on the object constraints, e.g. class and attributes names, associations and invariants, gives a very solid ground reinforced by object oriented programming environments (e.g. editor, compiler). With knowledge of the target information model and its built in constraints, it is possible to validate the Archetypes against the given model and prevent any erroneous constraints being created, which otherwise would cause object creation failure during runtime. Also the burden of binding constraints to the internal object model is no longer on the target systems, thus the degree of ambiguity is greatly reduced. This will in turn improve the interoperability among target EHR systems. Because the Archetypes are further constraints on the object models, it is possible to express constraints with general semantics of object models, e.g. existences, cardinalities and occurrences of attributes.

In Julius the Variables and Templates definitions are identified by their names, which are unique and meaningful to human users. This is sufficient in small scale usage, but will not be easy to scale up. For instance, different health care units could define different Julius Variables for the same medical concept, and if only the concept name is used as an identifier for the Julius Variables, namespace clashes are likely to happen. Also currently the naming of different version of the same Variables or Templates is done ad hoc – the Variables or Templates are simply given new names without any explicit rules. The openEHR Archetypes have formal rules for identification, which consists of the originator, the target model, the concept name and the version id. The combination of these sub-components should make the Archetype namespace much more maintainable.

Authoring of Julius Variables and Templates are done with the web based applications, CDS Admin and TDS Admin respectively. The results are directly stored on the sever side and later published by web services in XML format. That means there is no need for a human user to deal with interchangeable formats of any kind. There is also no need for a specific linguistic format parser because the binding from XML to object format is automated by Java XML Binding on the server side. So the whole chain of authoring, storing, sharing, parsing are fully integrated. From this aspect, the tooling support of openEHR Archetypes is not as thorough as that of Julius Variables and Templates even if the Julius ones are not as ambitious. There are currently GUI tools for editing and validating Archetypes and it is now possible to save Archetypes in either ADL or XML format. Although there are also parsers that can handle Archetypes in ADL format, there is not yet any server side component available for versioning, storing and publishing archetypes.

Table 1 summarizes the comparison between the Julius and openEHR models.

### Possible future improvements

Currently there is no versioning support for the definitions of the Julius Variables and Templates. Changes made to the definitions overwrite the previous ones, which might have been used for data entry already. If the changes are related to validation rules and data structures, it leads to that the previous record cannot be loaded with new definitions. One proposal is therefore to replace the current template and variable engine with the openEHR archetype based EHR kernel which has good versioning support.

The support of more data types and data structures is desirable for the improvement of expressiveness of the present Julius Variables and Templates. Without proper support for common data types and data structures, the user's ability to model clinical concept in the form of Variables and Templates is limited. It may lead to creation of poor Variables and Templates that do not reflect the real clinical need faithfully, which in turn might cause low quality of patient data. The way to improve this is to introduce more data types, e.g. ISO 11404 data types [9] and common data structures, such as List and Table, also available in openEHR.

With the openEHR model, the Julius user could benefit from the expressiveness of the openEHR reference model and also interoperability with other standard compliant EHR systems. In addition to interoperability using the full openEHR specifications, the function to export and import XML extracts in EN13606 format for patient data exchange between different EHR systems will become very useful in any EHR system.

A further development of Julius CDS has been proposed with the aim to provide direct access to well known reference terminologies such as ICD10 and SNOMED-CT by a separate concepts and terminology service that may also be accessible to non-Julius systems and to a Julius PDS application at runtime. A more advanced terminology binding design would be required to allow several reference terms to be associated with a Variable and value set items. Such separate terminology services will also be desirable in an archetype based system both when defining archetypes and sometimes at EHR runtime.

## Conclusion

The Julius system has met its design goals. The approach of the Julius system in combination with various local EHR systems can facilitate the sharing and reuse of validated clinical information from different health care units. However, future system developments for these purposes should consider using the openEHR/CEN models with shareable archetypes.

## Competing interests

The author(s) declare that they have no competing interests.

## Authors' contributions

Enberg contributed to the original design of the system, the project management of development and deployment. He also contributed with review and revision of this manuscript. Chen contributed to the improvement of the system with the new architectural design and performed the implementation of the software system and acted as the main drafter of the manuscript. Klein was serving as a senior advisor on the architectural design and contributed to the analysis comparing the Julius approach with the openEHR model. All authors have read and approved the final manuscript.

## Pre-publication history

The pre-publication history for this paper can be accessed here:



## Supplementary Material

Additional file 1 This archive contains all the source code files required to build the Julius system.Refer to the README.TXT file for an overview of the different files of the archive. Click here for file

## Figures and Tables

**Table 1:** Comparison of Julius PDS to openEHR Reference Information Model

**RM Features**	**Julius**	**openEHR**
data types	string, integer, decimal, date	Boolean, State, Identifier, Text, Coded Text, Paragraph, Count, Interval, Ordinal, Quantity, Quantity Ratio, Multimedia, Parsable, URI, Date, Time, Datetime, Duration
data structures – spatial	Group	Item Single, Item List, Item Tree, Item Table
data structures – history	N/A	History, Single Event, Event Series
Demographic	N/A	Party, Actor, Person,Organisation, Agent Address, Contact etc
object identification	Counters of type big integer	Object ID, Archetype ID, Terminology ID, Hierarchical Object ID, ISO OID, UID, UUID, Object Ref, Party Ref etc
change control	Version	Version, Contribution,Audit Details, Version Repository, Versioned Compositions, Directory
