# Supplementary material for: Julius – a template based supplementary electronic health record system
Source: BMC Med Inform Decis Mak. 2007 May 2;7:10. doi: 10.1186/1472-6947-7-10 (PMC1868711; doi:10.1186/1472-6947-7-10)
Supplement: Additional file 1 — This archive contains all the source code files required to build the Julius system.Refer to the README.TXT file for an overview of the different files of the archive. [file 1472-6947-7-10-S1.tgz › julius-src/cds.tgz!/cds/doc/ToB_admin_req_spec_v1-0.pdf]

Requirement Specifications for the ToB AdminTool

G Enberg
R Chen

Created On: 2003-7-5
Last Modified: 2003-7-8

Introduction

The administrative tool for Term and Concept Service (aka ToB) is to ease the maintenance of certified usage of terms in a regulated way. It consists of following modules:

- Term, ValueSet definition module
- Category based navigation
- Search function
- Role base access control
- User authentication and management

Entities in the Application

- Term: definition of a usage of a standard term.
- ValueSet: list of valid value items allowed as input to a term.
- Category: definition for a hierarchical tree-like structure where Terms and ValueSets will be found. It provides a way of navigating and organising of Terms and ValueSets.
- User: user profiles for authentication and authorization

User Roles

The application will have two type of users: Term Author and ToB Administrator with different level of rights and user stories.

Term Author

- create new Term and ValueSet
- modify non-active Term and ValueSet
- delete Term and ValueSet created(never activated)

ToB Administrator

- create, modify, delete Category
- create, modify and delete user profiles
- activate, deactivate Term and ValueSet

Status of Term and ValueSet

There are distinguished status for each Term And ValueSet entities:

- ***CREATED*** means just created by Term Author. It can be modified and deleted by the author, but can't be used within a template in the Template AdminTool.

- ***ACTIVE*** means approved by ToB Administrator and ready to be used in templates.
- ***INACTIVE*** means deactivated and thus not allowed to be used in templates.
- ***MODIFIED*** means the term has been modified by the author after it's deactivated

The transition of status is described in following diagram:

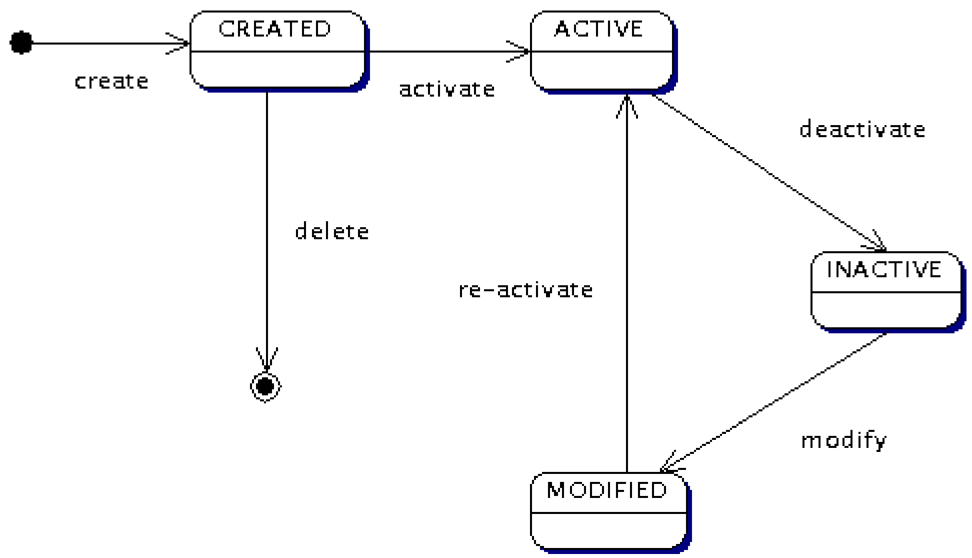

Term Use Cases

1. Creation

Information should be provided by the Term author during the creation of a Term.

Source	Source terminology from which the term is from
Referenceterm	The ID used in source terminology
Name	The unique name to be used as a reference in the DMS applications
Label	The text to be displayed on the user interface
FieldType	Text, Date, Numeric, ValueSet and etc.
Valueset *	The name of the ValueSet if the type is ValueSet
Category **	The name of the category where the term will reside
Description	Further information regarding the term

Other information that will be stored during creation:

- Status of the term will be ***CREATED*** after creation of the term.
- The creation timestamp will be saved.
- The creator of the Term definition

NOTE:

* ValueSet name should be provide by the system as drop-down list, probably a list of all ValueSet in the current category

** Category name should be provided by the category navigation system, probably use the name of current category

2. Modification

A Term can be modified by a Term Author when it's in ***INACTIVE*** status. The user who makes the modification, the reason and the timestamp of modification will be saved to the Term.

Name and FieldType are not allowed to be changed. The status will be ***MODIFIED*** after modification is done.

3. Deletion

Only when the Term has NEVER been activated, can it be deleted by its creator or ToB Administrator. When deleted, the definition will be removed from the database.

4. Activation/Deactivation

Only ToB Administrator can activate and deactivate a Term definition. The actual user, timestamp and reason will be recorded.

5. History Tracking

ToB Administrator should be able to view the history of a Term definition. That is a listing of all events(creation, modification, activation and inactivation) including the user, reason and timestamp.

ValueSet Use Cases

1. Creation

Information should be provided by the Term author during the creation of a ValueSet.

Name	The unique name to be used as a reference
Category *	The name of the category where the term will reside
Description	Further information regarding the term
Items **	List of allowed values

Other information that will be stored during creation:

- Status of the term will be *CREATED* after creation of the ValueSet.
- The creation timestamp will be saved.
- The creator of the ValueSet

NOTE:

* Category name should be provided by the category navigation system, probably use the name of current category

** Term Author should be able to insert/modify allowed value items without leaving the same user interface.

2. Modification

A ValueSet can be modified by a Term Author when it's in ***INACTIVE*** status. The user who makes the modification, the reason and the timestamp of modification will be saved.

Term Author should be able to add, remove allowed value items. Name can't be modified. The status will be ***MODIFIED*** after modification is done.

3. Deletion

Only when the ValueSet has NEVER been activated, can it be deleted by its creator or ToB Administrator. When deleted, the definition will be removed from the database.

4. Activation/Deactivation

Only ToB Administrator can activate and deactivate a ValueSet definition. The actual administrator, timestamp and reason will be recorded.

5. History Tracking

ToB Administrator should be able to view the history of a ValueSet definition. That is a listing of all events(creation, modification, activation and inactivation) including its user, reason and timestamp.

Category Use Cases

Only ToB Administrator is allowed to create, modify and delete category definitions.

1. Creation

Following information should be provided during creation of the category:

- Name: unique name in current level
- Parent category: current category implied
- Description: further information in text

Creator, timestamp will be recorded.

2. Modification

Name and description are possible to be updated.

3. Deletion

Category can only be deleted if there is no Term or ValueSet associated to it.

4. Navigation

The categories should be presented as a tree-like structure with clickable category names on the user interface. When a category is clicked by the user, it becomes the current category and used as the category for creating Term and ValueSet. The system should also provide a list of Terms and ValueSets associated to current category and it' s sub-categories. A click on listed Terms and ValueSets should lead user to the definition page.

User Management Use Cases

Only ToB administrator is allowed to create, update and delete user profiles.

1. User Creation

During creation of a User profile, following information should be provided:

- username
- password
- type: either Term Author or Administrator

After creation, the user should be able to log on system using created profile.

2. User Modification

username shouldn' t be modified. Term Author could be allowed to change his/her own password.

3. User Deletion

After deletion, the user shouldn' t be able to log on to the system using deleted user profile.

Navigation and Search System

The System should provide both category based navigation and search system so that the user can easily find a Term or ValueSet.

Search functions

When searching for Terms and ValueSet the following search criteria should be possible to use

- Full or partial name (of the term)
- Name of the category where the terms belongs to

- Date (of creation)
- Status

The result list should leads to the definition page of a Term or ValueSet.

Non-functional requirements

Platform and Standard

The server-side solution should only use standard J2EE components (JSP/Servlet), and should be deployable to any J2EE compliant application server.
The client-side solution should be limited to pure HTML page with Javascript so it can be run with mainstream browsers.

Design layout

Should follow the look-and-feel design for the administrative tool for templates.

Source Repository

All source code, documents and libraries used in build process should be stored in the single source repository provided by the project.

Testing

1. **Acceptance Test**
   The final production code has to pass the acceptance test provided by the project to be considered complete in functionality.
2. **Unit Testing**
   JUnit based unit tests for all major logic in business layer should be delivered together with production code. The execution of the tests should be integrated into the build process using Ant build script.

Documentation

1. **System Documentation**
   A concise and up-to-date system document should be provided. It will serve the developer who will maintain and extend the system.
2. **User Manual**
   A well-written manual to the tool is required. The audience will be end users, both ToB Administrator and Term Author.
3. **JavaDoc**
   JavaDoc styled documentation should be provided in the source code for all non-private methods and classes.
