# Supplementary material for: Julius – a template based supplementary electronic health record system
Source: BMC Med Inform Decis Mak. 2007 May 2;7:10. doi: 10.1186/1472-6947-7-10 (PMC1868711; doi:10.1186/1472-6947-7-10)
Supplement: Additional file 1 — This archive contains all the source code files required to build the Julius system.Refer to the README.TXT file for an overview of the different files of the archive. [file 1472-6947-7-10-S1.tgz › julius-src/cds.tgz!/cds/web/webpages/admin_category_frameset.jsp]

<% se.sllki.tob.utilities.WebApplicationInformation wai =
(se.sllki.tob.utilities.WebApplicationInformation) application.getAttribute("wai"); %>

.: CDS Admin :.

?controller=CategoryNavigator" scrolling="AUTO" marginwidth="4" marginheight="0">
?controller=AdminContent" scrolling="auto" marginwidth="4" marginheight="0">
<body>
<p>To view this page, you need a browser that supports frames.</p>
</body>
