# Supplementary material for: Julius – a template based supplementary electronic health record system
Source: BMC Med Inform Decis Mak. 2007 May 2;7:10. doi: 10.1186/1472-6947-7-10 (PMC1868711; doi:10.1186/1472-6947-7-10)
Supplement: Additional file 1 — This archive contains all the source code files required to build the Julius system.Refer to the README.TXT file for an overview of the different files of the archive. [file 1472-6947-7-10-S1.tgz › julius-src/cds.tgz!/cds/web/webpages/admin_category_navigator.jsp]

<%@ page import="java.util.\*,
se.sllki.tob.utilities.Tokens" %>
<%@ page import="se.sllki.tob.helpers.\*" %>
<% se.sllki.tob.utilities.WebApplicationInformation wai =
(se.sllki.tob.utilities.WebApplicationInformation) application.getAttribute("wai"); %>


/admin.css" >
.: CDS Admin :.

?controller=CategoryNavigatorSubmit" method="POST" target="content">


Kategorier

<% if(userObject.getRole().isAllowedToCreateCategory()){ %>
<% } %>
