# Supplementary material for: Julius – a template based supplementary electronic health record system
Source: BMC Med Inform Decis Mak. 2007 May 2;7:10. doi: 10.1186/1472-6947-7-10 (PMC1868711; doi:10.1186/1472-6947-7-10)
Supplement: Additional file 1 — This archive contains all the source code files required to build the Julius system.Refer to the README.TXT file for an overview of the different files of the archive. [file 1472-6947-7-10-S1.tgz › julius-src/cds.tgz!/cds/web/webpages/admin_edit_user.jsp]

<% se.sllki.tob.utilities.WebApplicationInformation wai =
(se.sllki.tob.utilities.WebApplicationInformation) application.getAttribute("wai"); %>
<%@ page import="se.sllki.tob.admin.\*" %>
<%@ page import="se.sllki.tob.beans.\*" %>
<%@ page import="java.util.\*" %>
<% UserBean editUser = (UserBean)request.getAttribute("editUser"); %>

/admin.css" >
.: CDS Admin :.

?controller=EditUserSubmit" method="POST" target="content">
<% String login = editUser.getLogin(); %>
<% if(login == null || login.length() < 1){
login = "Ny Användare - ej sparad";
} else {
login = "Användare: " + editUser.getLogin();
} %>

|  |  |  |
| --- | --- | --- |
| **<%= login %>** | | |
|  | | |
<% if(request.getAttribute("operationError") != null){ %>| <%= request.getAttribute("operationError") %> | | |
<% } %>|  | | |
| Användarnamn: | <% if(userObject.getObjectID() == null){ %> <% } else { %> <% } %> | <%= editUser.getLoginValidation() %> |
| Lösenord: |  | <%= editUser.getPasswordValidation() %> |
| Roll: | <% String oldRole = editUser.getRole(); %> <% Role[] roles = Role.ROLES; %> <% String role; %> <% for(int i = 0; i < roles.length; i++) { %> <% role = roles[i].getName(); %> > <%= roles[i].getLabel() %> <% } %> | <%= editUser.getRoleValidation() %> |
| Förnamn: |  | <%= editUser.getFirstNameValidation() %> |
| Efternamn: |  | <%= editUser.getLastNameValidation() %> |
| Email: |  | <%= editUser.getEmailValidation() %> |
| Enhet: |  | <%= editUser.getUnitValidation() %> |
| Status: | Inactive: > Active: > |  |
