# Supplementary material for: Julius – a template based supplementary electronic health record system
Source: BMC Med Inform Decis Mak. 2007 May 2;7:10. doi: 10.1186/1472-6947-7-10 (PMC1868711; doi:10.1186/1472-6947-7-10)
Supplement: Additional file 1 — This archive contains all the source code files required to build the Julius system.Refer to the README.TXT file for an overview of the different files of the archive. [file 1472-6947-7-10-S1.tgz › julius-src/cds.tgz!/cds/web/webpages/admin_edit_user_password.jsp]

<% se.sllki.tob.utilities.WebApplicationInformation wai =
(se.sllki.tob.utilities.WebApplicationInformation) application.getAttribute("wai"); %>
<%@ page import="se.sllki.tob.admin.\*" %>
<%@ page import="se.sllki.tob.beans.\*" %>
<%@ page import="java.util.\*" %>

/admin.css" >
.: CDS Admin :.

?controller=EditUserPasswordSubmit" method="POST" target="\_self">
<% String login = userObject.getUserBean().getLogin(); %>

|  |  |  |
| --- | --- | --- |
| **<%= login %>** | | |
|  | | |
| <% if(request.getAttribute("passwordUpdated") != null){ %> Lösenordet har ändrats! <% } %> | | |
|  | | |
| Gammalt lösenord: |  | <% if(request.getAttribute("validationException") != null){ %> <% HashMap validationException = (HashMap)request.getAttribute("validationException"); %> <% if(validationException.get("oldPassword") != null){ %> <%= validationException.get("oldPassword") %> <% } %> <% } %> |
| Nytt lösenord: |  | <% if(request.getAttribute("validationException") != null){ %> <% HashMap validationException = (HashMap)request.getAttribute("validationException"); %> <% if(validationException.get("newPassword1") != null){ %> <%= validationException.get("newPassword1") %> <% } %> <% } %> |
| Verifiera nytt lösenord: |  |  |
