# Supplementary material for: Julius – a template based supplementary electronic health record system
Source: BMC Med Inform Decis Mak. 2007 May 2;7:10. doi: 10.1186/1472-6947-7-10 (PMC1868711; doi:10.1186/1472-6947-7-10)
Supplement: Additional file 1 — This archive contains all the source code files required to build the Julius system.Refer to the README.TXT file for an overview of the different files of the archive. [file 1472-6947-7-10-S1.tgz › julius-src/cds.tgz!/cds/web/webpages/admin_header.jsp]

<%@ page import="se.sllki.tob.admin.\*" %>
<%@ page import="se.sllki.tob.utilities.\*" %>
<% se.sllki.tob.utilities.WebApplicationInformation wai = (se.sllki.tob.utilities.WebApplicationInformation)application.getAttribute("wai"); %>


/admin.css" >
.: CDS Admin :.
<%
String defaultSelected = "";
String editCategorySelected = "";
String editUserSelected = "";
String editUserPasswordSelected = "";
if(userObject.getPerformingAction() != null){
if(userObject.getPerformingAction().equals(User.EDIT\_CATEGORY)){
editCategorySelected = "selected";
}else if(userObject.getPerformingAction().equals(User.EDIT\_USER)){
editUserSelected = "selected";
}else if(userObject.getPerformingAction().equals(User.EDIT\_USER\_PASSWORD)){
editUserPasswordSelected = "selected";
}
}else{
defaultSelected = "selected";
}
%>
/admin\_bkg\_header.gif">
?controller=AdminHeaderSubmit" method="POST" target="admin\_content\_frameset">

|  |  |  |  |
| --- | --- | --- | --- |
| **<%= (String)request.getAttribute("headerText")%>** | /search.gif" border="0"> Sök | Logga ut | > [Välj] > Kategori <% if(userObject.getRole().isAllowedToUpdateUser()){ %> > Användare <% } %> > Ändra lösenord |
