# Supplementary material for: Julius – a template based supplementary electronic health record system
Source: BMC Med Inform Decis Mak. 2007 May 2;7:10. doi: 10.1186/1472-6947-7-10 (PMC1868711; doi:10.1186/1472-6947-7-10)
Supplement: Additional file 1 — This archive contains all the source code files required to build the Julius system.Refer to the README.TXT file for an overview of the different files of the archive. [file 1472-6947-7-10-S1.tgz › julius-src/cds.tgz!/cds/web/webpages/admin_list_history.jsp]

<% se.sllki.tob.utilities.WebApplicationInformation wai = (se.sllki.tob.utilities.WebApplicationInformation)application.getAttribute("wai"); %>
<%@ page import="se.sllki.tob.beans.\*" %>
<%@ page import="se.sllki.tob.admin.\*" %>
<%@ page import="java.util.\*" %>

/admin.css">
.: CDS Admin :.
<% List historyList = (List)request.getAttribute("historyList"); %>

|  |  |  |  |
| --- | --- | --- | --- |
| **Historik f�r: <%= request.getParameter("name") %>** | | | |
|  | | | |
| Status | Orsak | Utf�rt av | Tid |
<% Iterator iter = historyList.iterator(); %>
<% History history = null; %>
<% while (iter.hasNext()){ %>
<% history = (History)iter.next(); %>| <%= Status.getStatusText(history.getStatus()) %> | <%= history.getReason() %> | <%= history.getSavedBy() %> | <%= history.getSavedTime() %> |
<% } %>
