# Supplementary material for: Julius – a template based supplementary electronic health record system
Source: BMC Med Inform Decis Mak. 2007 May 2;7:10. doi: 10.1186/1472-6947-7-10 (PMC1868711; doi:10.1186/1472-6947-7-10)
Supplement: Additional file 1 — This archive contains all the source code files required to build the Julius system.Refer to the README.TXT file for an overview of the different files of the archive. [file 1472-6947-7-10-S1.tgz › julius-src/cds.tgz!/cds/web/webpages/admin_search.jsp]

<% se.sllki.tob.utilities.WebApplicationInformation wai = (se.sllki.tob.utilities.WebApplicationInformation)application.getAttribute("wai"); %>
<%@ page import="java.util.\*" %>
<%@ page import="se.sllki.tob.admin.\*" %>
<%@ page import="se.sllki.tob.helpers.\*" %>

<%
//Obtain Today's Date
Calendar lo\_Cal = Calendar.getInstance();
int Day = lo\_Cal.get(Calendar.DAY\_OF\_MONTH);
int Month = lo\_Cal.get(Calendar.MONTH) + 1;
int Year = lo\_Cal.get(Calendar.YEAR);
String ls\_Month = String.valueOf(Month);
String ls\_Day = String.valueOf(Day);
if (Month < 10){
ls\_Month = "0" + ls\_Month;
}
if (Day < 10){
ls\_Day = "0"+ls\_Day;
}
String ls\_Today = String.valueOf(Year)+"-"+ls\_Month+"-"+ls\_Day;
%>
<%
HashMap searchArguments = search.getSearchArguments();
Object objectType = searchArguments.get("objectType");
Object category = searchArguments.get("category");
//Object referenceTerm = searchArguments.get("referenceTerm");
Object name = searchArguments.get("name");
Object status = searchArguments.get("status");
Object date = searchArguments.get("date");
Object dateFrom = searchArguments.get("dateFrom");
Object dateTo = searchArguments.get("dateTo");
%>

/admin.css">
.: CDS Admin :.

?controller=SearchSubmit" method="POST" target="content" onSubmit="return validate()">

|  |  |  |  |
| --- | --- | --- | --- |
| **Sök** | | | |
|  |  |  |  |
|  | | | |
| Typ: | > Term  > Värdeset  > Term och Värdeset |  |  |
| Kategori: | "> |  |  |
<%--| Term: | "> |  |  |
--%>| Namn: | "> |  |  |
| Status: | <% TreeMap statusList = new TreeMap(Status.getStatusTypes()); %> <% String statusID; %> <% Iterator iter = statusList.keySet().iterator(); %> <% while(iter.hasNext()){ %> <% statusID = (String)iter.next(); %> > <%= (String)statusList.get(statusID) %> <% } %> |  |  |
| Exakt Datum: | " readonly> | /calender.gif" width="22" height="21" border="0">  Töm fält |  |
| Från Datum: | " readonly> | /calender.gif" width="22" height="21" border="0">  Töm fält |  |
| Till Datum: | " readonly> | /calender.gif" width="22" height="21" border="0">  Töm fält |  |
| **Sökresultat** | | |  |
|  | | |  |
| Namn | | Typ | |
<% Iterator searchIter = search.getSearchList().iterator(); %>
<% SearchListObject sLO; %>
<% while (searchIter.hasNext()){ %>
<% sLO = (SearchListObject)searchIter.next(); %>| ?controller=SearchSubmit&objectID=<%= sLO.getObjectName() %>&objectType=<%= sLO.getObjectType() %>&categoryID=<%= sLO.getCategoryID() %>&clientCommand=OPEN" target="content" class="bluelink"> <%= sLO.getObjectName() %> | | <%= sLO.getObjectType().equals("T") ? "Term" : "Värdeset" %> | |
<% } %>
