# Supplementary material for: Julius – a template based supplementary electronic health record system
Source: BMC Med Inform Decis Mak. 2007 May 2;7:10. doi: 10.1186/1472-6947-7-10 (PMC1868711; doi:10.1186/1472-6947-7-10)
Supplement: Additional file 1 — This archive contains all the source code files required to build the Julius system.Refer to the README.TXT file for an overview of the different files of the archive. [file 1472-6947-7-10-S1.tgz › julius-src/cds.tgz!/cds/web/webpages/exception.jsp]

<%@ page isErrorPage="true" %>
<%@ page import="se.sllki.tob.utilities.\*" %>
<% se.sllki.tob.utilities.WebApplicationInformation wai = (se.sllki.tob.utilities.WebApplicationInformation)application.getAttribute("wai"); %>


Exception


/exception.css" >
/client\_general.css" >


Julius felmeddelande:

<%
Exception e = null;
if (exception != null) {
e = (Exception)exception;
} else if (request.getAttribute("Exception") != null) {
e = (Exception)request.getAttribute("Exception");
} else if (session.getAttribute("Exception") != null) {
e = (Exception)session.getAttribute("Exception");
} else {
e = new Exception("Unknown Exception");
}
ExtendedException ee = null;
if (e instanceof ExtendedException) {
ee = (ExtendedException)e;
} else {
ee = new ExtendedException(false, e.getMessage());
}
session.setAttribute("Exception", ee);
%>
<%
out.println("|  |
| --- |
| "); out.println(""); out.println(" " + ee + ", message:" + ee.getMessage()); out.println("  "); out.println(""); out.println(" |
");
%>
<%
java.util.Iterator i = ee.getMesaures().iterator();
while (i.hasNext()) {
ExtendedExceptionMesure eem = (ExtendedExceptionMesure)i.next();
out.println("| "); out.println(""); out.println(""); out.println("( " + eem.description + " )"); out.println(""); out.println(" |
");
}
String startURL = wai.getAppInfo("ADMINISTRATION\_SERVLET\_URI");
out.println("| "); out.println(""); out.println(""); out.println("( G� tillbaka till startsidan )"); out.println(""); out.println(" |
");
%>
<%
//out.println("| "); //out.println(" "); //out.println(""); //e.printStackTrace(new PrintWriter(out)); //out.println(""); //out.println(" |
");
%>
