# Supplementary material for: Julius – a template based supplementary electronic health record system
Source: BMC Med Inform Decis Mak. 2007 May 2;7:10. doi: 10.1186/1472-6947-7-10 (PMC1868711; doi:10.1186/1472-6947-7-10)
Supplement: Additional file 1 — This archive contains all the source code files required to build the Julius system.Refer to the README.TXT file for an overview of the different files of the archive. [file 1472-6947-7-10-S1.tgz › julius-src/cdsadmin.tgz!/cdsadmin/web/webpages/admin_user_navigator.jsp]

<% se.sllki.tob.utilities.WebApplicationInformation wai = (se.sllki.tob.utilities.WebApplicationInformation)application.getAttribute("wai"); %>
<%@ page import="se.sllki.tob.utilities.\*" %>
<%@ page import="se.sllki.tob.beans.\*" %>
<%@ page import="se.sllki.tob.helpers.\*" %>
<%@ page import="java.util.\*" %>

/admin.css">
.: CDS Admin :.

?controller=UserNavigatorSubmit" method="POST" target="content">

Anv�ndare

<% if(request.getAttribute("users") != null){ %>
<% ArrayList users = (ArrayList)request.getAttribute("users"); %>
<% if(users != null){ %>
<% Iterator iter = users.iterator(); %>
<% while(iter.hasNext()){ %>
<% UserBean user = (UserBean)iter.next(); %>
?controller=UserNavigatorSubmit&userID=<%= user.getLogin() %>&clientCommand=OPEN" target="content" class="bluelink">
<%= user.getLogin() %> [<%= user.getRole() %>]<%= user.isActive() ? "" : ", Ej aktiv" %>
  
<% } %>
<% } %>
<% } %>
