# Supplementary material for: Julius – a template based supplementary electronic health record system
Source: BMC Med Inform Decis Mak. 2007 May 2;7:10. doi: 10.1186/1472-6947-7-10 (PMC1868711; doi:10.1186/1472-6947-7-10)
Supplement: Additional file 1 — This archive contains all the source code files required to build the Julius system.Refer to the README.TXT file for an overview of the different files of the archive. [file 1472-6947-7-10-S1.tgz › julius-src/pds.tgz!/pds/test/res/totalReport.html]

Total Report


# Total Report

|  | | Doctor's Patients | | Unit's Patients | |
| --- | --- | --- | --- | --- | --- |
|  | | Total | % | Total | % |
| Total Patient |  | 10 | 100 | 40 | 100 |
|  | | | | | |
| Gender | male | 6 | 60% | 12 | 30% |
|  | female | 4 | 40% | 28 | 70% |
|  | | | | | |
| Age Range | 0-29y | 4 | 40% | 10 | 25% |
|  | 30-59y | 1 | 10% | 6 | 15% |
|  | 60- y | 5 | 50% | 24 | 60% |
|  | | | | | |
| valueset | yes | 2 | 20% | 18 | 45% |
|  | no | 4 | 40% | 12 | 30% |
|  | unknown | 4 | 40% | 10 | 25% |
|  | | | | | |
| numeric | <1.7 | 2 | 33% | 4 | 40% |
|  | >=1.7 | 4 | 67% | 6 | 60% |
|  | | | | | |
