# Supplementary material for: Julius – a template based supplementary electronic health record system
Source: BMC Med Inform Decis Mak. 2007 May 2;7:10. doi: 10.1186/1472-6947-7-10 (PMC1868711; doi:10.1186/1472-6947-7-10)
Supplement: Additional file 1 — This archive contains all the source code files required to build the Julius system.Refer to the README.TXT file for an overview of the different files of the archive. [file 1472-6947-7-10-S1.tgz › julius-src/pds.tgz!/pds/web/error.jsp]

<%@ page isErrorPage="true" %>
PDS - Error Message


# Dear user, regarding your last action:

The action you wished to execute generated an internal error
for some reason. Please hit the Back button to try again.

If this does not work or should you have any questions or comments,
please send a short description of the problem to:
G�sta Enberg gosta.enberg@nlpo.sll.se

We apologize for any inconvenience and will make every
effort to assit you as soon as possible
