# Supplementary material for: Julius – a template based supplementary electronic health record system
Source: BMC Med Inform Decis Mak. 2007 May 2;7:10. doi: 10.1186/1472-6947-7-10 (PMC1868711; doi:10.1186/1472-6947-7-10)
Supplement: Additional file 1 — This archive contains all the source code files required to build the Julius system.Refer to the README.TXT file for an overview of the different files of the archive. [file 1472-6947-7-10-S1.tgz › julius-src/pds.tgz!/pds/web/webpages/dmx_applet.jsp]

<%@ page isErrorPage="false" %>
<%@ page errorPage="/webpages/exception.jsp" %>
<% se.sllki.julius.web.utilities.WebApplicationInfo wai =
(se.sllki.julius.web.utilities.WebApplicationInfo)application.getAttribute("wai"); %>

/dmx\_applet.css">


/SDMX.jar">

?controller=DmxRequest" >
