# Supplementary material for: Julius – a template based supplementary electronic health record system
Source: BMC Med Inform Decis Mak. 2007 May 2;7:10. doi: 10.1186/1472-6947-7-10 (PMC1868711; doi:10.1186/1472-6947-7-10)
Supplement: Additional file 1 — This archive contains all the source code files required to build the Julius system.Refer to the README.TXT file for an overview of the different files of the archive. [file 1472-6947-7-10-S1.tgz › julius-src/pds.tgz!/pds/web/webpages/main_frameset.jsp]

<%@ page isErrorPage="false" %>
<%@ page errorPage="/webpages/exception.jsp" %>
<%@ page import="se.sllki.julius.web.utilities.\*,
se.sllki.pds.web.Tokens" %>
<% WebApplicationInfo wai = (WebApplicationInfo)application.getAttribute("wai"); %>

?controller=MainMenu" frameborder="0" marginheight="0" marginwidth="0">
?controller=MainContent" frameborder="0" marginheight="0" marginwidth="0">
