# Supplementary material for: Julius – a template based supplementary electronic health record system
Source: BMC Med Inform Decis Mak. 2007 May 2;7:10. doi: 10.1186/1472-6947-7-10 (PMC1868711; doi:10.1186/1472-6947-7-10)
Supplement: Additional file 1 — This archive contains all the source code files required to build the Julius system.Refer to the README.TXT file for an overview of the different files of the archive. [file 1472-6947-7-10-S1.tgz › julius-src/pds.tgz!/pds/web/webpages/patients_search_menu.jsp]

<%@ page isErrorPage="false" %>
<%@ page errorPage="/webpages/exception.jsp" %>
<%@ page import="java.io.\*" %>
<%@ page import="java.util.\*" %>
<%@ page import="se.sllki.julius.utilities.\*" %>
<%@ page import="se.sllki.julius.web.utilities.\*" %>
<%@ page import="se.sllki.pds.proxy.\*" %>
<% ////User user = (User)session.getAttribute("user"); %>
<% //VisitBean visitBean = (VisitBean)session.getAttribute("patientBean"); %>
<% //HashMap createPatientErrors = (HashMap)request.getAttribute("createPatientErrors"); %>
<% se.sllki.julius.web.utilities.WebApplicationInfo wai = (se.sllki.julius.web.utilities.WebApplicationInfo) application.getAttribute("wai"); %>
<% ApplicationData applicationData = (ApplicationData)application.getAttribute("applicationData"); %>

/patients\_search\_menu.css" >
/general.css" >

?controller=PatientsSearchMenuSubmit" method="POST">

Patienter

|  |  |  |  |
| --- | --- | --- | --- |
| Personnummer |  | Diagnos | <% renderDropdown("diagnosis","choiceListName", out, applicationData); %> |
|  | | | |

<%!
// -----------------------------------------------------------------------------
// --- renderErrors()
// -----------------------------------------------------------------------------
void renderErrors(String uniqueFieldIdentifier, JspWriter out, TemplateFieldMetaDataElement field, HashMap visitErrors) throws IOException{
if (visitErrors != null && !visitErrors.isEmpty()) {
String error = (String)visitErrors.get(field.getName());
if (error != null) {
uniqueFieldIdentifier = uniqueFieldIdentifier + "-ERROR";
if (field.getEditable() == 1) {
out.println("");
} else {
out.println("");
}
}
}
}
%>
<%!
// -----------------------------------------------------------------------------
// --- renderDropdown()
// -----------------------------------------------------------------------------
void renderDropdown(String uniqueFieldIdentifier, String choiceListName, JspWriter out, ApplicationData applicationData) throws Exception {
out.println("");
out.println("");
try {
TemplateServiceProxy templateService = new TemplateServiceProxy();
String[] templates = templateService.findTemplateNames();
for(int i = 0; i < templates.length; i++) {
out.println(""
+ templates[i] + "");
}
} catch(Exception e) {
e.printStackTrace();
}
out.println("");
}
%>
