# Supplementary material for: Julius – a template based supplementary electronic health record system
Source: BMC Med Inform Decis Mak. 2007 May 2;7:10. doi: 10.1186/1472-6947-7-10 (PMC1868711; doi:10.1186/1472-6947-7-10)
Supplement: Additional file 1 — This archive contains all the source code files required to build the Julius system.Refer to the README.TXT file for an overview of the different files of the archive. [file 1472-6947-7-10-S1.tgz › julius-src/pds.tgz!/pds/web/webpages/patients_search_result.jsp]

<%@ page isErrorPage="false" %>
<%@ page errorPage="/webpages/exception.jsp" %>
<%@ page import="java.util.\*" %>
<%@ page import="se.sllki.julius.utilities.\*" %>
<%@ page import="se.sllki.julius.web.utilities.\*" %>
<% se.sllki.julius.web.utilities.WebApplicationInfo wai = (se.sllki.julius.web.utilities.WebApplicationInfo)this.getServletContext().getAttribute("wai"); %>
<% PatientsSearchResult pSR = (PatientsSearchResult)request.getAttribute("patientsSearchResult"); %>
<% String diagnosisId = (String)request.getAttribute("diagnosisId"); %>

/general.css" >
/patients\_search\_result.css" >


S�kresultat

  
<%
if (pSR.size() > 0) {
Iterator i = pSR.patientsSearchResultIterator();
PatientsSearchResultItem pSRI = null;
out.print("

");
String link = null;
while(i.hasNext()){
pSRI = (PatientsSearchResultItem)i.next();
link = wai.getAppInfo("APPLICATION\_SERVLET\_URI") + "?controller=PatientVisitsList&patientId=" + pSRI.getPatientId() + "&diagnosisId=" + diagnosisId;
out.print("|  |
| --- |
| " + pSRI.getPatientNumber() + " |
");
}
} else {
out.print("inga poster att visa");
}
%>
