# Supplementary material for: Julius – a template based supplementary electronic health record system
Source: BMC Med Inform Decis Mak. 2007 May 2;7:10. doi: 10.1186/1472-6947-7-10 (PMC1868711; doi:10.1186/1472-6947-7-10)
Supplement: Additional file 1 — This archive contains all the source code files required to build the Julius system.Refer to the README.TXT file for an overview of the different files of the archive. [file 1472-6947-7-10-S1.tgz › julius-src/pds.tgz!/pds/web/webpages/patient_visits_list.jsp]

<%@ page isErrorPage="false" %>
<%@ page errorPage="/webpages/exception.jsp" %>
<%@ page import="java.util.\*" %>
<%@ page import="se.sllki.julius.utilities.\*" %>
<%@ page import="se.sllki.julius.web.utilities.\*" %>
<% se.sllki.julius.web.utilities.WebApplicationInfo wai = (se.sllki.julius.web.utilities.WebApplicationInfo)this.getServletContext().getAttribute("wai"); %>
<% PatientVisitsSearchResult pVSR = (PatientVisitsSearchResult)request.getAttribute("patientVisitsSearchResult"); %>

/general.css" >
/patient\_visits\_list.css" >


<%
if (pVSR.size() > 0) {
out.print("Besöklista för: " + pVSR.getPatientNumber());
} else {
out.print("Besöklista för: < ingen >");
}
%>

  
<%
if (pVSR.size() > 0) {
Iterator i = pVSR.patientVisitsSearchResultIterator();
PatientVisitsSearchResultItem pVSRI = null;
out.print("

");
String link = null;
while(i.hasNext()){
pVSRI = (PatientVisitsSearchResultItem)i.next();
link = wai.getAppInfo("APPLICATION\_SERVLET\_URI") + "?controller=OpenVisitTemplate&visitId=" + pVSRI.getVisitId() + "&patientId=" + pVSR.getPatientId();
out.print("|  |
| --- |
| " + pVSRI.getVisitDate() + " |
");
}
} else {
out.print("inga besök att visa");
}
%>
