# Supplementary material for: Julius – a template based supplementary electronic health record system
Source: BMC Med Inform Decis Mak. 2007 May 2;7:10. doi: 10.1186/1472-6947-7-10 (PMC1868711; doi:10.1186/1472-6947-7-10)
Supplement: Additional file 1 — This archive contains all the source code files required to build the Julius system.Refer to the README.TXT file for an overview of the different files of the archive. [file 1472-6947-7-10-S1.tgz › julius-src/pds.tgz!/pds/web/webpages/visittemplate_content.jsp]

<%@ page isErrorPage="false" %>
<%@ page errorPage="/webpages/exception.jsp" %>
<%@ page import="java.io.\*,
se.sllki.pds.proxy.soap.\*,
se.sllki.pds.proxy.\*,
se.sllki.pds.web.Tokens" %>
<%@ page import="java.util.\*" %>
<%@ page import="se.sllki.julius.utilities.\*" %>
<%@ page import="se.sllki.julius.web.utilities.\*" %>
<% WebApplicationInfo wai = (WebApplicationInfo) application.getAttribute("wai"); %>
<% // disabled for demo // VisitBean visitBean = (VisitBean)session.getAttribute("visitBean"); %>
<% HashMap visitErrors = (HashMap)session.getAttribute("visitErrors"); %>
<% Template template = (Template)session.getAttribute(Tokens.SES\_TEMPLATE\_BEAN); %>

/visittemplate\_content.css" >
/general.css" >

?controller=VisitTemplateContentSubmit" method="POST">
<%
renderTemplate(out, request, visitErrors, template);
// todo: disabled for demo
// renderTemplate(out, request, visitBean, visitErrors, template);
%>

<%
String value = "FALSE";
String clientUnsavedVisitData = (String)request.getSession().getAttribute("clientUnsavedVisitData");
if (clientUnsavedVisitData != null) {
if (clientUnsavedVisitData.equalsIgnoreCase("TRUE")) {
value = "TRUE";
}
}
%>
">

">
">
<%!
// -----------------------------------------------------------------------------
// --- renderTemplate()
// -----------------------------------------------------------------------------
void renderTemplate(JspWriter out, HttpServletRequest request,
//VisitBean visitBean,
HashMap visitErrors,
Template template) throws Exception {
int currentGroupIndex = 0;
try {
currentGroupIndex = Integer.parseInt(
(String)request.getAttribute(Tokens.REQ\_CURRENT\_GROUP\_INDEX));
} catch(NumberFormatException ignored) {}
boolean displayGroupHeader = false;
if(currentGroupIndex == -1) {
displayGroupHeader = true;
}
GroupType group = null;
for(int i = 0, y = template.getGroup().size(); i < y; i++) {
group = (GroupType) template.getGroup().get(i);
if ( currentGroupIndex == -1 || currentGroupIndex == i) {
renderGroup(out, group, //todo: visitBean,
displayGroupHeader, i,
template.getName(), visitErrors);
}
}
}
%>
<%!
// -----------------------------------------------------------------------------
// --- renderGroup()
// -----------------------------------------------------------------------------
void renderGroup(JspWriter out, GroupType group, //VisitBean visitBean,
boolean displayGroupHeader, int groupIndex,
String templateIndex, HashMap visitErrors) throws Exception {
Iterator fields = group.getField().iterator();
FieldType field = null;
Term term = null;
VisitDataElement data = null;
String visitIndex = ""; // todo: visitBean.getVisitId().toString();
if (displayGroupHeader) {
out.println("

");
out.println("|  |
| --- |
|");
out.println(" "); out.println(group.getName()); out.println(" |");
out.println("
");
out.println("

");
}
out.println("

");
int fieldIndex = 0;
String uniqueFieldIdentifier = "";
while (fields.hasNext()) {
TermServiceProxy termService = new TermServiceProxy();
field = (FieldType)fields.next();
term = termService.findTerm(field.getName());
uniqueFieldIdentifier = "T" + templateIndex + "V" + visitIndex + "G" + groupIndex + "F" + fieldIndex;
// todo: disabled for demo
//data = (VisitDataElement)visitBean.getVisitData().get(field.getName());
if (data == null) {
data = new VisitDataElement();
}
out.println("|  |  |  |  |
| --- | --- | --- | --- |
|");
out.println(" "); String uFI = uniqueFieldIdentifier + "-INFORMATION"; /\* nytt fönster \*/ out.println(""); out.println(" |");
out.println(" "); renderLabel(uniqueFieldIdentifier, out, field, term); out.println(" |");
out.println(" "); int type = Integer.parseInt(term.getType()); if (type == 5) { renderDropdown(uniqueFieldIdentifier, out, field, data, term); } else if (type == 2) { renderNumericField(uniqueFieldIdentifier, out,field, data, term); } else if (type == 1) { renderTextField(uniqueFieldIdentifier, out,field, data, term); } else if (type == 3) { renderDateField(uniqueFieldIdentifier, out,field, data, term); } else if (type == 7) { renderTextArea(uniqueFieldIdentifier, out,field, data, term); } else if (type == 4) { renderCheckBox(uniqueFieldIdentifier, out,field, data, term); } else if (type == 8) { renderBasicScoreField(uniqueFieldIdentifier, out,field, data, term); } out.println(" |");
out.println(" "); renderFielddateField(uniqueFieldIdentifier, out, field, data, term); renderUnit(uniqueFieldIdentifier, out, field, term); renderPeriodWaring(uniqueFieldIdentifier); renderErrors(uniqueFieldIdentifier, out, field, visitErrors, term); out.println(" |");
out.println("
");
fieldIndex++;
}
out.println("

");
}
%>
<%!
// -----------------------------------------------------------------------------
// --- renderLabel()
// -----------------------------------------------------------------------------
void renderLabel(String uniqueFieldIdentifier, JspWriter out, FieldType field,
Term term) throws IOException{
uniqueFieldIdentifier = uniqueFieldIdentifier + "-LABEL";
if (field.isEditable()) {
if (field.isGraph()) {
out.println("");
}
out.println("" + term.getLabel() + "");
if (field.isGraph()) {
out.println("");
}
} else {
if (field.isGraph()) {
out.println("");
}
out.println("" + term.getLabel() + "");
if (field.isGraph()) {
out.println("");
}
}
}
%>
<%!
// -----------------------------------------------------------------------------
// --- renderFielddateField()
// -----------------------------------------------------------------------------
void renderFielddateField(String uniqueFieldIdentifier, JspWriter out,
FieldType field, VisitDataElement data, Term term)
throws IOException{
uniqueFieldIdentifier = uniqueFieldIdentifier + "-FIELDDATE";
if (field.isShowDate()) {
String value = (data.getDate() != null && data.getDate().length() > 0 ) ? data.getDate() : "";
if (field.isEditable()) {
out.println("");
} else {
out.println("");
}
}
}
%>
<%!
// -----------------------------------------------------------------------------
// --- renderUnit()
// -----------------------------------------------------------------------------
void renderUnit(String uniqueFieldIdentifier, JspWriter out, FieldType field,
Term term) throws IOException{
uniqueFieldIdentifier = uniqueFieldIdentifier + "-UNIT";
String unit = (term.getUnit() != null && term.getUnit().length() > 0 ) ? term.getUnit() : " ";
if (field.isEditable()) {
out.println("" + unit + "");
} else {
out.println("" + unit + "");
}
}
%>
<%!
// -----------------------------------------------------------------------------
// --- renderPeriodWaring()
// -----------------------------------------------------------------------------
void renderPeriodWaring(String uniqueFieldIdentifier) throws IOException{
uniqueFieldIdentifier = uniqueFieldIdentifier + "-PERIOD";
//if (field.getUpdatePeriod() != "") {
/\*
int days = Integer.parseInt(field.getUpdatePeriod()) \* 30;
Calendar calendar = Calendar.getInstance();
Date today = calendar.getTime();
if (today > calendar.add(calendar.DAY\_OF\_YEAR, -1 \* days)) {
} else if (today > calendar.add(calendar.DAY\_OF\_YEAR, (int)days/2)) {
}
\*/
/\*
if (field.getEditable().equalsIgnoreCase(field.YES.toString())) {
out.println("");
} else {
out.println("");
}
\*/
//}
}
%>
<%!
// -----------------------------------------------------------------------------
// --- renderErrors()
// -----------------------------------------------------------------------------
void renderErrors(String uniqueFieldIdentifier, JspWriter out,
FieldType field, HashMap visitErrors, Term term) throws IOException{
if (visitErrors != null && !visitErrors.isEmpty()) {
String error = (String)visitErrors.get(field.getName());
if (error != null) {
uniqueFieldIdentifier = uniqueFieldIdentifier + "-ERROR";
if (field.isEditable()) {
out.println("");
} else {
out.println("");
}
}
}
}
%>
<%!
// -----------------------------------------------------------------------------
// --- renderDropdown()
// -----------------------------------------------------------------------------
void renderDropdown(String uniqueFieldIdentifier, JspWriter out,
FieldType field, VisitDataElement data, Term term)
throws Exception {
uniqueFieldIdentifier = uniqueFieldIdentifier + "-DROPDOWN";
if (field.isEditable()) {
out.println("");
out.println("");
ValueSetType valueSet = term.getValueSet();
if (valueSet != null) {
Iterator i = valueSet.getItem().iterator();
String selectedValue = (data.getValue() != null && data.getValue().length() > 0 ) ? data.getValue() : "-1";
while (i.hasNext()) {
ItemType item = (ItemType)i.next();
if (item.getValue().equalsIgnoreCase(selectedValue)) {
out.println("" + item.getLabel() + "");
} else {
out.println("" + item.getLabel() + "");
}
}
}
out.println("");
} else {
out.println("");
LinkedList choiceList = new LinkedList();
//applicationData.getChoiceList(field.getCodetable());
if (choiceList != null) {
Iterator i = choiceList.iterator();
CodeTableElement cLE;
String selectedValue = (data.getValue() != null && data.getValue().length() > 0 ) ? data.getValue() : "-1";
while (i.hasNext()) {
cLE = (CodeTableElement)i.next();
if (cLE.getCode().toString().trim().equalsIgnoreCase(selectedValue)) {
out.println("" + cLE.getDescription() + "");
}
}
}
out.println("");
}
}
%>
<%!
// -----------------------------------------------------------------------------
// --- renderCheckBox()
// -----------------------------------------------------------------------------
void renderCheckBox(String uniqueFieldIdentifier, JspWriter out, FieldType field,
VisitDataElement data, Term term) throws IOException{
uniqueFieldIdentifier = uniqueFieldIdentifier + "-CHECKBOX";
String value = (data.getValue() != null && data.getValue().length() > 0 ) ? data.getValue() : "0";
if (field.isEditable()) {
if (Integer.parseInt(value) == 1) {
out.println("");
} else {
out.println("");
}
} else {
if (Integer.parseInt(value) == 1) {
out.println("");
} else {
out.println("");
}
}
}
%>
<%!
// -----------------------------------------------------------------------------
// --- renderDateField()
// -----------------------------------------------------------------------------
void renderDateField(String uniqueFieldIdentifier, JspWriter out,
FieldType field, VisitDataElement data, Term term)
throws IOException{
uniqueFieldIdentifier = uniqueFieldIdentifier + "-DATEFIELD";
String value = (data.getValue() != null && data.getValue().length() > 0 ) ? data.getValue() : "";
//value = new JuliusDateFormat().getDateWithSeparator(value);
//value = (value != null && value.length() > 0) ? value:"";
if (field.isEditable()) {
String validate = (field.isShallBeValidated()) ? "true" : "false";
out.println("");
} else {
out.println("");
}
}
%>
<%!
// -----------------------------------------------------------------------------
// --- renderTextArea()
// -----------------------------------------------------------------------------
void renderTextArea(String uniqueFieldIdentifier, JspWriter out,
FieldType field, VisitDataElement data, Term term)
throws IOException{
uniqueFieldIdentifier = uniqueFieldIdentifier + "-TEXTAREA";
String value = (data.getValue() != null && data.getValue().length() > 0 ) ? data.getValue() : "";
if (field.isEditable()) {
String validate = (field.isShallBeValidated()) ? "true" : "false";
out.println("" + value + "");
} else {
out.println("" + value + "");
}
}
%>
<%!
// -----------------------------------------------------------------------------
// --- renderTextField()
// -----------------------------------------------------------------------------
void renderTextField(String uniqueFieldIdentifier, JspWriter out,
FieldType field, VisitDataElement data, Term term)
throws IOException{
uniqueFieldIdentifier = uniqueFieldIdentifier + "-TEXTFIELD";
String value = (data.getValue() != null && data.getValue().length() > 0 ) ? data.getValue() : "";
if (field.isEditable()) {
String validate = (field.isShallBeValidated()) ? "true" : "false";
out.println("");
} else {
out.println("");
}
}
%>
<%!
// -----------------------------------------------------------------------------
// --- renderNumericField()
// -----------------------------------------------------------------------------
void renderNumericField(String uniqueFieldIdentifier,
JspWriter out, FieldType field, VisitDataElement data,
Term term)
throws IOException{
uniqueFieldIdentifier = uniqueFieldIdentifier + "-NUMERICFIELD";
String value = (data.getValue() != null && data.getValue().length() > 0 ) ? data.getValue() : "";
if (field.isEditable()) {
String validate = (field.isShallBeValidated()) ? "true" : "false";
out.println("");
} else {
out.println("");
}
}
%>
<%!
// -----------------------------------------------------------------------------
// --- renderBasicScoreField()
// -----------------------------------------------------------------------------
void renderBasicScoreField(String uniqueFieldIdentifier, JspWriter out,
FieldType field, VisitDataElement data, Term term)
throws IOException{
uniqueFieldIdentifier = uniqueFieldIdentifier + "-BASICSCOREFIELD";
String value = (data.getValue() != null && data.getValue().length() > 0 ) ? data.getValue() : "";
//if (field.isEditable()) {
// String validate = (field.isShallBeValidated()) ? "true" : "false";
// out.println("");
//} else {
out.println("");
//}
}
%>
