# Supplementary material for: Julius – a template based supplementary electronic health record system
Source: BMC Med Inform Decis Mak. 2007 May 2;7:10. doi: 10.1186/1472-6947-7-10 (PMC1868711; doi:10.1186/1472-6947-7-10)
Supplement: Additional file 1 — This archive contains all the source code files required to build the Julius system.Refer to the README.TXT file for an overview of the different files of the archive. [file 1472-6947-7-10-S1.tgz › julius-src/pds.tgz!/pds/web/webpages/visittemplate_header.jsp]

<%@ page isErrorPage="false" %>
<%@ page errorPage="/webpages/exception.jsp" %>
<%@ page import="java.io.\*,
se.sllki.pds.web.Tokens" %>
<%@ page import="java.util.\*" %>
<%@ page import="java.text.\*" %>
<%@ page import="se.sllki.julius.businesslogic.\*" %>
<%@ page import="se.sllki.julius.utilities.\*" %>
<%@ page import="se.sllki.julius.web.utilities.\*" %>
<%@ page import="se.sllki.pds.proxy.soap.Template" %>
<% WebApplicationInfo wai = (WebApplicationInfo)application.getAttribute("wai"); %>
<%
// todo: disabled for demo
// PatientBean patientBean = (PatientBean)session.getAttribute("patientBean");
%>
<% //User user = (User)session.getAttribute("user"); %>
<% //VisitBean visitBean = (VisitBean)session.getAttribute("visitBean"); %>
<% HashMap visitErrors = (HashMap)session.getAttribute("visitErrors"); %>
<% Template template = (Template)session.getAttribute(Tokens.SES\_TEMPLATE\_BEAN); %>

/visittemplate\_header.css" >
/general.css" >


<% // todo: hardcoded value //patientBean.getPatientFormattedNumber() %>
<%= "12121212-1212" %>
  

<% //= patientBean.getPatientName() %>
<% // todo: disabled
/\*String patientname = patientBean.getPatientName();
if (patientname.length() > 25) {
patientname = patientname.substring(0,25);
}
out.print(patientname);\*/
%>

Status: <% //renderTemplateStatus(out, visitBean, visitErrors); %>

<%= template.getName() %>

<%
// todo: dirty fix for demo
if (VisitStatus.STATUS\_APPROVED != null) {
//if ((visitBean.getStatus().compareTo(VisitStatus.STATUS\_APPROVED) == 0)) {
// || (user.isUserInRole(applicationData.ROLE\_READER))) {
%>
stäng
hjälp
skriv ut
<%
} else {
%>
godkänn
spara utkast
stäng
radera
hjälp
skriv ut
<% } %>

Datum: <% // todo %> <% renderTemplateDateField(out);// renderTemplateDateField(out, visitBean); %>

<% // todo: diabled for demo
//renderVisitBrowseButtons(out, visitBean, patientBean); %>

<% // todo: disabled for demo
// renderPatientVisitsDropdown(out, visitBean, patientBean); %>

<%!
// -----------------------------------------------------------------------------
// --- renderPatientVisitsDropdown()
// -----------------------------------------------------------------------------
void renderPatientVisitsDropdown(JspWriter out, VisitBean visitBean, PatientBean patientBean) throws IOException{
out.println("");
Iterator i = patientBean.getVisits().iterator();
String visitDate;
String visitId;
Date tempDate = null;
String status = null;
String text = null;
while (i.hasNext()) {
VisitListElement visitListElement = (VisitListElement)i.next();
visitId = visitListElement.getVisitId().toString();
try {
tempDate = new SimpleDateFormat("yyyyMMdd").parse(visitListElement.getVisitDate());
visitDate = new SimpleDateFormat("yyyy'-'MM'-'dd").format(tempDate);
} catch (ParseException e) {
visitDate = "";
}
status = (String) new VisitStatus().getStatusCollection().get(visitListElement.getVisitStatus());
text = visitDate + " : " + status;
if (visitBean.getVisitId().compareTo( new Integer(visitId)) == 0) {
out.println("" + text + "");
} else {
out.println("" + text + "");
}
}
out.println("");
}
%>
<%!
// -----------------------------------------------------------------------------
// --- renderTemplateStatus()
// -----------------------------------------------------------------------------
void renderTemplateStatus(JspWriter out, VisitBean visitBean, HashMap visitErrors) throws IOException{
String templateStatus = (String)new VisitStatus().getStatusCollection().get(visitBean.getStatus());
if (visitErrors != null && !visitErrors.isEmpty()) {
templateStatus = (String)new VisitStatus().getStatusCollection().get(VisitStatus.STATUS\_NOTSAVED);
out.print("");
String message = "Besök : ogiltiga värden.\\n";
String temp = (String)visitErrors.get("visitDate");
if (temp != null) {
message = message + "\\n" + temp;
}
message = message + "\\n\\n Ett eller flera värden är ogiltiga.";
out.println("");
} else {
out.print("");
}
}
%>
<%!
// -----------------------------------------------------------------------------
// --- renderTemplateDateField()
// -----------------------------------------------------------------------------
// todo: changed for demo
//void renderTemplateDateField(JspWriter out, VisitBean visitBean) throws IOException{
void renderTemplateDateField(JspWriter out) throws IOException{
//String date = visitBean.getDate();
String date = DateFormat.getInstance().format(new Date());
out.println("");
}
%>
<%!
// -----------------------------------------------------------------------------
// --- renderVisitBrowseButtons()
// -----------------------------------------------------------------------------
void renderVisitBrowseButtons(JspWriter out, VisitBean visitBean, PatientBean patientBean) throws IOException {
String currentVisitId = visitBean.getVisitId().toString();
LinkedList visits = patientBean.getVisits();
VisitListElement vLE = null;
int index = 0;
int noOfVisits = visits.size();
for(int i = 0; i < noOfVisits; i++) {
vLE = (VisitListElement)visits.get(i);
if (vLE.getVisitId().toString().equalsIgnoreCase(currentVisitId)) {
index = i+1;
break;
}
}
if (index > 1) {
out.print("");
out.print("");
} else {
out.print("");
out.print("");
}
out.print("");
if (index < noOfVisits) {
out.print("");
out.print("");
} else {
out.print("");
out.print("");
}
}
%>
