# Supplementary material for: Julius – a template based supplementary electronic health record system
Source: BMC Med Inform Decis Mak. 2007 May 2;7:10. doi: 10.1186/1472-6947-7-10 (PMC1868711; doi:10.1186/1472-6947-7-10)
Supplement: Additional file 1 — This archive contains all the source code files required to build the Julius system.Refer to the README.TXT file for an overview of the different files of the archive. [file 1472-6947-7-10-S1.tgz › julius-src/pds.tgz!/pds/web/webpages/visittemplate_help.jsp]

<%@ page isErrorPage="false" %>
<%@ page errorPage="/webpages/exception.jsp" %>
<%@ page import="java.util.\*" %>
<%@ page import="se.sllki.julius.utilities.\*" %>
<%@ page import="se.sllki.julius.web.utilities.\*" %>
<% WebApplicationInfo wai = (WebApplicationInfo)application.getAttribute("wai"); %>
<% LinkedList helpText = (LinkedList)request.getAttribute("helpText"); %>

.:Julius:.


/visittemplate\_help.css" >

skriv ut
<% Iterator iter = helpText.iterator(); %>
<% while(iter.hasNext()){ %>
<% HelpTextElement helpTextElement = (HelpTextElement)iter.next(); %>
<% if (helpTextElement.getType().equalsIgnoreCase("TEMPLATE")){ %>

Hj�lp f�r mall : <%= helpTextElement.getName() %>

<% } %>
<% if (helpTextElement.getType().equalsIgnoreCase("TEMPLATE")){ %>

<%= helpTextElement.getText() %>

<% } %>
<% if (helpTextElement.getType().equalsIgnoreCase("GROUP")){ %>

Flik: <%= helpTextElement.getName() %>

<%= helpTextElement.getText() %>

<% } %>

<% if (helpTextElement.getType().equalsIgnoreCase("FIELD")){ %>|  |
| --- |
| <%= helpTextElement.getName() %>: |
| <%= helpTextElement.getText() %> |
<% } %>

<% } %>
