# Supplementary material for: Julius – a template based supplementary electronic health record system
Source: BMC Med Inform Decis Mak. 2007 May 2;7:10. doi: 10.1186/1472-6947-7-10 (PMC1868711; doi:10.1186/1472-6947-7-10)
Supplement: Additional file 1 — This archive contains all the source code files required to build the Julius system.Refer to the README.TXT file for an overview of the different files of the archive. [file 1472-6947-7-10-S1.tgz › julius-src/pds.tgz!/pds/web/webpages/visittemplate_menu.jsp]

<%@ page isErrorPage="false" %>
<%@ page errorPage="/webpages/exception.jsp" %>
<%@ page import="java.io.\*,
se.sllki.pds.proxy.soap.Template,
se.sllki.pds.proxy.soap.GroupType,
se.sllki.pds.web.Tokens" %>
<%@ page import="java.util.\*" %>
<%@ page import="se.sllki.julius.utilities.\*" %>
<%@ page import="se.sllki.julius.web.utilities.\*" %>
<% WebApplicationInfo wai = (WebApplicationInfo)application.getAttribute("wai"); %>
<%
Template template = (Template)session.getAttribute(Tokens.SES\_TEMPLATE\_BEAN);
application.log("### template: " + template.getName() + ", groups:" + template.getGroup().size());
%>

/visittemplate\_menu.css" >


<% renderGroupMenu(out, request, template); %>
<%!
// -----------------------------------------------------------------------------
// --- renderGroupMenu()
// -----------------------------------------------------------------------------
private void renderGroupMenu(JspWriter out, HttpServletRequest request,
Template template) throws Exception {
int currentGrp = 0;
try {
currentGrp = Integer.parseInt(
(String)request.getAttribute(Tokens.REQ\_CURRENT\_GROUP\_INDEX));
} catch(NumberFormatException ignored) {
}
int index = 0;
GroupType group = null;
for (Iterator it = template.getGroup().iterator();it.hasNext();) {
group = (GroupType) it.next();
out.println("");
}
out.println("

---

");
out.println("");
//boolean visitErrors = (request.getSession().getAttribute("visitErrors") != null) ? true : false;
//if (visitErrors) {
// if ( Integer.parseInt(currentGroupIndex) == -2) {
// out.println("");
// } else {
// out.println("");
// }
//}
}
%>
