# Supplementary material for: Julius – a template based supplementary electronic health record system
Source: BMC Med Inform Decis Mak. 2007 May 2;7:10. doi: 10.1186/1472-6947-7-10 (PMC1868711; doi:10.1186/1472-6947-7-10)
Supplement: Additional file 1 — This archive contains all the source code files required to build the Julius system.Refer to the README.TXT file for an overview of the different files of the archive. [file 1472-6947-7-10-S1.tgz › julius-src/pds.tgz!/pds/web/webpages/_visitlist_result.jsp]

<%@ page isErrorPage="false" %>
<%@ page errorPage="/webpages/exception.jsp" %>
<%@ page import="java.util.\*" %>
<%@ page import="se.sllki.julius.utilities.\*" %>
<%@ page import="se.sllki.julius.businesslogic.\*" %>
<% se.sllki.julius.web.utilities.WebApplicationInfo wai = (se.sllki.julius.web.utilities.WebApplicationInfo)this.getServletContext().getAttribute("wai"); %>
<%
HashMap status = new VisitStatus().getStatusCollection();
%>

/\_visitlist.css" >


|  |  |  |  |
| --- | --- | --- | --- |
| Bes�ksdatum | Patient | Status | Diagnos |
<% LinkedList results = (LinkedList)request.getAttribute("searchResults"); %>
<% Iterator i = results.listIterator(); %>
<% HashMap temp = null; %>
<% boolean bToggle = true; %>
<% while (i.hasNext()) { %>
<% temp = (HashMap)i.next(); %>
<% if (bToggle == true) { %>|<% bToggle = false; %>
<% } else { %>|  |  |  |  |
| --- | --- | --- | --- |
|<% bToggle = true; %>
<% } %> ?controller=\_OpenVisit&patientid=<%= temp.get("patientId") %>&visitid=<%= temp.get("visitId") %>" TARGET="top\_content"> <%= JuliusDateFormat.getDateWithSeparator((String)temp.get("visitDate")) %> | <%= temp.get("patientNumber") %> | <%= status.get(new Integer((String)temp.get("statusId"))) %> | <%= temp.get("templateName") %> |
<% } %>  |  |  |  |
