# Supplementary material for: Julius – a template based supplementary electronic health record system
Source: BMC Med Inform Decis Mak. 2007 May 2;7:10. doi: 10.1186/1472-6947-7-10 (PMC1868711; doi:10.1186/1472-6947-7-10)
Supplement: Additional file 1 — This archive contains all the source code files required to build the Julius system.Refer to the README.TXT file for an overview of the different files of the archive. [file 1472-6947-7-10-S1.tgz › julius-src/tds.tgz!/tds/src/web/admin/toc.html]

List all services

Start a service

Stop a service
