# Supplementary material for: Julius – a template based supplementary electronic health record system
Source: BMC Med Inform Decis Mak. 2007 May 2;7:10. doi: 10.1186/1472-6947-7-10 (PMC1868711; doi:10.1186/1472-6947-7-10)
Supplement: Additional file 1 — This archive contains all the source code files required to build the Julius system.Refer to the README.TXT file for an overview of the different files of the archive. [file 1472-6947-7-10-S1.tgz › julius-src/tds.tgz!/tds/src/web/webpages/admin_displayvariablelist.jsp]

<% se.sllki.julius.web.utilities.WebApplicationInformation wai =
(se.sllki.julius.web.utilities.WebApplicationInformation) application.getAttribute("wai"); %>
<%@ page import="se.sllki.julius.utilities.\*,
java.util.List" %>

/admin.css">
.: Julius :.

?controller=ViewVariableList" method="POST" target="variables\_content"><% String search = (String)request.getAttribute("search"); %>

<% String[] variablesFirstCases = (String[])request.getAttribute("variablesFirstCases"); %>
<% for(int i=0;i
<% String firstCase = variablesFirstCases[i]; %>
?controller=ViewVariableList&search=<%= firstCase %>&resultsFrom=0" target="variables\_content" class="bluelink"><%= firstCase %>   <% } %>

Sök:

|  |  |
| --- | --- |
| <% if(request.getAttribute("variableNames") != null){ %> <% String[] variableNames = (String[])request.getAttribute("variableNames"); %> <% int totNumVariables = variableNames.length; %> <% if(variableNames.length > 0){ %> **<%= new Integer((String)request.getAttribute("resultsFrom")).intValue()+1 %> - <%= new Integer((String)request.getAttribute("resultsTo")).intValue()+1 %> (<%= variableNames.length %>)**  <% } %> |  |
|<% if(totNumVariables < 21){ %> <% for(int i=0;i <% String variableName = variableNames[i]; %>     ?controller=ViewVariableList&variable=<%= variableName %>&search=<%= search %>" target="variables\_content" class="bluelink"><%= variableName %>  <% } %> |  |
<% }else{ %>| <% List variableNamesShow = (List)request.getAttribute("variableNamesShow"); %> <% java.util.Iterator iterator3 = variableNamesShow.iterator(); %> <% int i = 0; %> <% while(iterator3.hasNext()){ %> <% String variableName = (String)iterator3.next(); %> <% if(i==20){ %> | <% } %> <% i++; %>     ?controller=ViewVariableList&variable=<%= variableName %>&search=<%= search %>" target="variables\_content" class="bluelink"><%= variableName %>  <% } %> |
| <% if(new Integer((String)request.getAttribute("resultsFrom")).intValue() > 1){ %>  föregående » <% } %> <% if(new Integer((String)request.getAttribute("resultsTo")).intValue()+1 < variableNames.length){ %>   nästa »  <% } %> |  |
<% } %>
<% } %>
