# Supplementary material for: Julius – a template based supplementary electronic health record system
Source: BMC Med Inform Decis Mak. 2007 May 2;7:10. doi: 10.1186/1472-6947-7-10 (PMC1868711; doi:10.1186/1472-6947-7-10)
Supplement: Additional file 1 — This archive contains all the source code files required to build the Julius system.Refer to the README.TXT file for an overview of the different files of the archive. [file 1472-6947-7-10-S1.tgz › julius-src/tds.tgz!/tds/src/web/webpages/admin_handlefield.jsp]

<%@ page import="se.sllki.julius.utilities.\*,
se.sllki.julius.binding.ValueSetType,
se.sllki.julius.admin.Highlight,
se.sllki.julius.web.utilities.Tokens" %>
<%@ page import="java.util.\*" %>
<% se.sllki.julius.web.utilities.WebApplicationInformation wai =
(se.sllki.julius.web.utilities.WebApplicationInformation) application.getAttribute("wai"); %>

/admin.css" >
editmetadata.jsp

?controller=HandleFieldSubmit" method="POST" target="\_parent">  
<% String templateId = (String) request.getAttribute("template"); %>
<% String groupId = (String)request.getAttribute("group"); %>
<% String fieldId = (String)request.getAttribute("field"); %>
<% FieldBean data = (FieldBean)request.getAttribute("fieldElement"); %>

|  |  |
| --- | --- |
|  | |
|  | |
|  | |
| Namn: | <%= data.getName() %> (<%= data.getId()%>) |
| Fälttyp: |  |
| Etikett: |  |
| Beskrivning: | <%= data.getDescription() %> |
| Enhet: |  |
<%--| Uppdateringsperiod: |  |
| Visa datum? | <% if(data.getShowDate().equals("1")){ %> Ja Nej <% }else{ %> Ja Nej <% } %> |
--%>
<% if (data.getValuesetName() != null) { %>| Kodtabell: |  |
<% } %>
<%--
<% if (!data.getFieldType().equals("8")) { %>| Kan värde editeras? | <% if(data.getEditable().equals("0")){ %> Ja Nej <% }else{ %> Ja Nej <% } %> |
<% } %>
<% if (!data.getFieldType().equals("4")) { %>| Kan värde saknas? | <% if(data.getMissing().equals("0")){ %> Ja Nej <% }else{ %> Ja Nej <% } %> |
<% } %>
<% if (!(data.getFieldType().equals("4") | data.getFieldType().equals("5"))) { %>| Valideras? | <% if(data.getShallBeValidated().equals("0")){ %> Ja Nej <% }else{ %> Ja Nej <% } %> |
<% } %>
<% if (!data.getFieldType().equals("4")) { %>| Checkas? | <% if(data.getShallBeChecked().equals("0")){ %> Ja Nej <% }else{ %> Ja Nej <% } %> |
<% } %>
<% if (!(data.getFieldType().equals("4") | data.getFieldType().equals("5"))) { %>| Checkregler: |  |
<% } %>
--%>
<% if (TemplateFieldType.DECIMAL.getName().equals(data.getFieldType())) { %>| Max (hård) |  |
| Min (hård): |  |
| Max (mjuk): |  |
| Min (mjuk): |  |
<%--| Max antal decimaler: |  |
--%>
<% } %>
<%-- <% if (data.getFieldType().equals("3")) { %>| Datum från: |  |
| Datum till: |  |
<% } %>
--%>
<%--
<% if (data.getFieldType().equals("1") | data.getFieldType().equals("7")) { %>| Max antal tecken: |  |
| Min antal tecken: |  |
<% } %>
<% if (data.getFieldType().equals("2") | data.getFieldType().equals("5") | data.getFieldType().equals("8")) { %>| Graf? | <% if(data.getGraph().equals("1")){ %> Ja Nej <% }else{ %> Ja Nej <% } %> |
<% } %>
--%>
| Highlight | <% Highlight[] lists = Highlight.HIGHLIGHT\_LIST; for(int i = 0; i < lists.length; i++) { %>  value="<%= lists[i].getId() %>"><%= lists[i].getLabel() %> <% } %> |
| Multiple Value Support | >Ja >Nej |
| Enter Value Once Only | >Ja >Nej |
<%-- <% if (data.getFieldType().equals("8")) { %>
<% Map templateFields = (Map)request.getAttribute("templateFields"); %>
<% Map depFields = (Map)request.getAttribute("depFields"); %>

|  |  |  |
| --- | --- | --- |
| Fields on template: |  | Dependents: |
| <% Iterator iter = templateFields.keySet().iterator(); %> <% while(iter.hasNext()){ %> <% Long id = (Long)iter.next(); %> <% if(!depFields.containsKey(id) && !id.equals(new Long(fieldId))){ %> <% String name = (String)templateFields.get(id); %> <%= name %> <% } %> <% } %> |  | <% Iterator iter2 = depFields.keySet().iterator(); %> <% while(iter2.hasNext()){ %> <% Long id = (Long)iter2.next(); %> <% String name = (String)templateFields.get(id); %> <%= name %> <% } %> |

<% } %>--%>
