# Supplementary material for: Julius – a template based supplementary electronic health record system
Source: BMC Med Inform Decis Mak. 2007 May 2;7:10. doi: 10.1186/1472-6947-7-10 (PMC1868711; doi:10.1186/1472-6947-7-10)
Supplement: Additional file 1 — This archive contains all the source code files required to build the Julius system.Refer to the README.TXT file for an overview of the different files of the archive. [file 1472-6947-7-10-S1.tgz › julius-src/tds.tgz!/tds/src/web/webpages/admin_handlefield_list.jsp]

<%@ page import="se.sllki.julius.utilities.\*,
java.util.List,
java.util.Iterator" %>
<% se.sllki.julius.web.utilities.WebApplicationInformation wai =
(se.sllki.julius.web.utilities.WebApplicationInformation)application.getAttribute("wai"); %>

/admin.css">
.: Julius :.
<% String templateId = (String)request.getAttribute("template"); %>
<% String groupId = (String)request.getAttribute("group"); %>
<% String fieldIndex = (String)request.getAttribute("fieldindex"); %>
?controller=HandleFieldListSubmit&template=<%= templateId %>&group=<%= groupId %>" method="POST">


?controller=HandleTemplate&template=<%= templateId %>&group=<%= groupId %>&field=new" target="\_parent" class="bluelink">
**Lägg till fält »**

**Välj fält:**

/admin\_arrow\_up.gif" border="0" onclick="setCommand('moveUp')">

<% List fields = (List)session.getAttribute("fields"); %>
<% Iterator iterator = fields.iterator(); %>
<% int i = 0; %>
<% int activeIndex = fieldIndex != null ? new Integer(fieldIndex).intValue() : -1; %>
<% while(iterator.hasNext()){ %>
<% FieldBean element = (FieldBean)iterator.next(); %>
<% if(activeIndex > -1 && i == activeIndex){ %>
?controller=HandleTemplate&template=<%= templateId %>&group=<%= groupId %>&field=<%= element.getId() %>');return document.MM\_returnValue"><%= element.getName() %>  
<% }else{ %>
?controller=HandleTemplate&template=<%= templateId %>&group=<%= groupId %>&field=<%= element.getId() %>');return document.MM\_returnValue"><%= element.getName() %>  
<% } %>
<% i++; %>
<% } %>

/admin\_arrow\_down.gif" border="0" onclick="setCommand('moveDown')">
