# Supplementary material for: Julius – a template based supplementary electronic health record system
Source: BMC Med Inform Decis Mak. 2007 May 2;7:10. doi: 10.1186/1472-6947-7-10 (PMC1868711; doi:10.1186/1472-6947-7-10)
Supplement: Additional file 1 — This archive contains all the source code files required to build the Julius system.Refer to the README.TXT file for an overview of the different files of the archive. [file 1472-6947-7-10-S1.tgz › julius-src/tds.tgz!/tds/src/web/webpages/admin_handlegroup.jsp]

<%@ page import="se.sllki.julius.utilities.\*" %>
<% se.sllki.julius.web.utilities.WebApplicationInformation wai = (se.sllki.julius.web.utilities.WebApplicationInformation)application.getAttribute("wai"); %>
<% String text = request.getParameter("group").equals("new") ? "Ny grupp" : "Redigera grupp"; %>
<% String templateId = request.getParameter("template"); %>
<% String groupId = request.getParameter("group"); %>
<% GroupBean groupElement = (GroupBean)request.getAttribute("groupElement"); %>

/admin.css" >
.: Julius :.

?controller=HandleGroupSubmit" method="POST" target="\_parent">
  
  

|  |  |
| --- | --- |
| **<%= text %>** | |
|  | |
|  | |
| Namn: |  |
|  | |
| Beskrivning: | <%= groupElement.getDescription() %> |
|  | |
|  | |
|  |  |
|  |  |
