# Supplementary material for: Julius – a template based supplementary electronic health record system
Source: BMC Med Inform Decis Mak. 2007 May 2;7:10. doi: 10.1186/1472-6947-7-10 (PMC1868711; doi:10.1186/1472-6947-7-10)
Supplement: Additional file 1 — This archive contains all the source code files required to build the Julius system.Refer to the README.TXT file for an overview of the different files of the archive. [file 1472-6947-7-10-S1.tgz › julius-src/tds.tgz!/tds/src/web/webpages/admin_handlegroup_addfield.jsp]

<%@ page import="se.sllki.julius.utilities.\*,
java.util.List" %>
<% se.sllki.julius.web.utilities.WebApplicationInformation wai = (se.sllki.julius.web.utilities.WebApplicationInformation)application.getAttribute("wai"); %>

/admin.css" >
.: Julius :.

?controller=HandleGroupAddFieldSubmit" method="POST" target="\_parent">
<% String templateId = (String)request.getAttribute("template"); %>
<% String groupId = (String)request.getAttribute("group"); %>
<% List variables = (List)request.getAttribute("variables"); %>
  
  

|  |  |
| --- | --- |
| **Lägg till fält** | |
|  | |
| Nytt fält: | <% for(int i = 0;i <% String name = (String)variables.get(i); %> <%= name %> <% } %> |
|  | |
|  |  |
|  |  |
