# Supplementary material for: Julius – a template based supplementary electronic health record system
Source: BMC Med Inform Decis Mak. 2007 May 2;7:10. doi: 10.1186/1472-6947-7-10 (PMC1868711; doi:10.1186/1472-6947-7-10)
Supplement: Additional file 1 — This archive contains all the source code files required to build the Julius system.Refer to the README.TXT file for an overview of the different files of the archive. [file 1472-6947-7-10-S1.tgz › julius-src/tds.tgz!/tds/src/web/webpages/admin_handlegroup_list.jsp]

<%@ page import="se.sllki.julius.utilities.\*,
java.util.List" %>
<% se.sllki.julius.web.utilities.WebApplicationInformation wai = (se.sllki.julius.web.utilities.WebApplicationInformation)application.getAttribute("wai"); %>

/admin.css" >
.: Julius :.
<% String templateId = (String)request.getAttribute("template"); %>
<% String groupIndex = (String)request.getAttribute("groupindex"); %>
?controller=HandleGroupListSubmit&template=<%= templateId %>" method="POST">


?controller=HandleTemplate&template=<%= templateId %>&group=new" target="\_parent" class="bluelink">
**Ny grupp »**

**Välj grupp:**

/admin\_arrow\_up.gif" border="0" onclick="setCommand('moveUp')">

<% List groups = (List)session.getAttribute("groups"); %>
<% java.util.Iterator iterator = groups.iterator(); %>
<% int i = 0; %>
<% int activeIndex = groupIndex != null ? new Integer(groupIndex).intValue() : -1; %>
<% while(iterator.hasNext()){ %>
<% GroupBean element = (GroupBean)iterator.next(); %>
<% if(activeIndex > -1 && i == activeIndex){ %>
?controller=HandleTemplate&template=<%= templateId %>&group=<%= element.getId() %>');return document.MM\_returnValue"><%= element.getName() %>  
<% }else{ %>
?controller=HandleTemplate&template=<%= templateId %>&group=<%= element.getId() %>');return document.MM\_returnValue"><%= element.getName() %>  
<% } %>
<% i++; %>
<% } %>

/admin\_arrow\_down.gif" border="0" onclick="setCommand('moveDown')">
