# Supplementary material for: Julius – a template based supplementary electronic health record system
Source: BMC Med Inform Decis Mak. 2007 May 2;7:10. doi: 10.1186/1472-6947-7-10 (PMC1868711; doi:10.1186/1472-6947-7-10)
Supplement: Additional file 1 — This archive contains all the source code files required to build the Julius system.Refer to the README.TXT file for an overview of the different files of the archive. [file 1472-6947-7-10-S1.tgz › julius-src/tds.tgz!/tds/src/web/webpages/admin_top_frameset.jsp]

<%@ page language="java" buffer="64kb" %>
<% se.sllki.julius.web.utilities.WebApplicationInformation wai = (se.sllki.julius.web.utilities.WebApplicationInformation)application.getAttribute("wai"); %>

?controller=MainFrameset" frameborder="0" marginheight="0" marginwidth="0">
