# Supplementary material for: Julius – a template based supplementary electronic health record system
Source: BMC Med Inform Decis Mak. 2007 May 2;7:10. doi: 10.1186/1472-6947-7-10 (PMC1868711; doi:10.1186/1472-6947-7-10)
Supplement: Additional file 1 — This archive contains all the source code files required to build the Julius system.Refer to the README.TXT file for an overview of the different files of the archive. [file 1472-6947-7-10-S1.tgz › julius-src/tds.tgz!/tds/src/web/webpages/admin_viewvariable.jsp]

<% se.sllki.julius.web.utilities.WebApplicationInformation wai = (se.sllki.julius.web.utilities.WebApplicationInformation) application.getAttribute("wai"); %>
<%@ page import="se.sllki.julius.binding.Term,
se.sllki.julius.binding.ValueSetType,
se.sllki.julius.binding.ItemType" %>
<%@ page import="se.sllki.julius.utilities.\*" %>
<%@ page import="java.util.\*" %>

/admin.css" >
.: Julius :.

?controller=ViewVariableSubmit" method="POST" target="variables\_content">
  
  
<% Term term = (Term)request.getAttribute("variableData"); %>

|  |  |
| --- | --- |
|  | |
|  | |
| **<%= term.getName() %>** | |
|  | |
|  | |
| Namn: | <%= term.getName() %> |
| Label: | <%= term.getLabel() %> |
| Fälttyp: | <%= term.getType() %> |
| Enhet: | <% if(term.getUnit() != null){ %> <%= term.getUnit() %> <% } %> |
|  | |
| Beskrivning: | <%= term.getDescription() %> |
|  | |

  
  
<%
ValueSetType valueSet = term.getValueSet();
if(valueSet != null) {
%>

|  |  |
| --- | --- |
|  | |
| **<%= valueSet.getName() %>** | |
<% List items = valueSet.getItem(); %>
<% boolean greyBg = false; %>
<%
for(Iterator it = items.iterator(); it.hasNext();){
ItemType item = (ItemType) it.next();
%>
<% if(greyBg){ %>|<% greyBg = false;
}else{ %>|  |  |
| --- | --- |
|<% greyBg = true;
} %> <%= item.getValue() %> | <%= item.getLabel() %> |
<% } %>|  |  |
  |  |

<%
}
%>
