# Supplementary material for: Julius – a template based supplementary electronic health record system
Source: BMC Med Inform Decis Mak. 2007 May 2;7:10. doi: 10.1186/1472-6947-7-10 (PMC1868711; doi:10.1186/1472-6947-7-10)
Supplement: Additional file 1 — This archive contains all the source code files required to build the Julius system.Refer to the README.TXT file for an overview of the different files of the archive. [file 1472-6947-7-10-S1.tgz › julius-src/tds.tgz!/tds/src/web/webpages/base_exception.jsp]

<%@ page import="se.sllki.julius.utilities.\*,
java.util.List" %>
<%@ page import="se.sllki.julius.web.utilities.\*" %>
<% se.sllki.julius.web.utilities.WebApplicationInformation wai = (se.sllki.julius.web.utilities.WebApplicationInformation)application.getAttribute("wai"); %>


Exception


/base\_exception.css" >
/client\_general.css" >


Julius felmeddelande:

<% JuliusBaseException exception = (JuliusBaseException)request.getAttribute("Exception"); %>
<% List exceptionMessages = exception.getMessages(); %>
<% for(int i = 0; i < exceptionMessages.size(); i++){ %>|  |
| --- |
| <%= exceptionMessages.get(i) %> |
<% } %>

<% if (exception.getUrl() != null) { %>
<% } %>
