# Supplementary material for: Julius – a template based supplementary electronic health record system
Source: BMC Med Inform Decis Mak. 2007 May 2;7:10. doi: 10.1186/1472-6947-7-10 (PMC1868711; doi:10.1186/1472-6947-7-10)
Supplement: Additional file 1 — This archive contains all the source code files required to build the Julius system.Refer to the README.TXT file for an overview of the different files of the archive. [file 1472-6947-7-10-S1.tgz › julius-src/tds.tgz!/tds/src/web/webpages/error.jsp]

<%@ page isErrorPage="true" %>
Exception


# Exception

#### An exception has occured while processing request: <%= request.getAttribute("sourceURL") %>

**Message:** <%= exception.getMessage() %>  
**Exception:** <%= exception %>
<% application.log((String)request.getAttribute("sourceURL"), exception); %>
